# Quantitative disentanglement of nanocrystalline phases in cement pastes by synchrotron ptychographic X-ray tomography

**DOI:** 10.1107/S2052252519003774

**Published:** 2019-04-27

**Authors:** Ana Cuesta, Ángeles G. De la Torre, Isabel Santacruz, Ana Diaz, Pavel Trtik, Mirko Holler, Barbara Lothenbach, Miguel A. G. Aranda

**Affiliations:** a Departamento de Química Inorgánica, Cristalografía y Mineralogía, Universidad de Málaga, 29071-Malaga, Spain; b Paul Scherrer Institut, 5232 Villigen PSI, Switzerland; c Faculty of Civil Engineering, Czech Technical University in Prague, 166 29 Prague, Czech Republic; d EMPA, Laboratory for Concrete and Construction Chemistry, Überlandstrasse 129, CH-8600 Dübendorf, Switzerland; e ALBA Synchrotron, Carrer de la Llum 2-26, E-08290 Cerdanyola del Vallès, Barcelona, Spain

**Keywords:** Portland cement, X-ray imaging, microstructure determination, density measurements, C-S-H gels, amorphous hydrogarnet, synchrotron ptychographic tomography, nanocrystalline components, thermodynamic modelling

## Abstract

Mortars and concretes are ubiquitous materials with very complex hierarchical microstructures. To fully understand their main properties and to decrease their CO_2_ footprint, a sound description of their spatially resolved mineralogy is necessary. Developing this knowledge is very challenging as about half of the volume of hydrated cement is a nanocrystalline component, calcium silicate hydrate (C-S-H) gel. Furthermore, other poorly crystalline phases (*e.g.* iron siliceous hydrogarnet or silica oxide) may coexist, which are even more difficult to characterize. Traditional spatially resolved techniques such as electron microscopy involve complex sample preparation steps that often lead to artefacts (*e.g.* dehydration and microstructural changes). Here, synchrotron ptychographic tomography has been used to obtain spatially resolved information on three unaltered representative samples: neat Portland paste, Portland–calcite and Portland–fly-ash blend pastes with a spatial resolution below 100 nm in samples with a volume of up to 5 × 10^4^ µm^3^. For the neat Portland paste, the ptychotomographic study gave densities of 2.11 and 2.52 g cm^−3^ and a content of 41.1 and 6.4 vol% for nanocrystalline C-S-H gel and poorly crystalline iron siliceous hydrogarnet, respectively. Furthermore, the spatially resolved volumetric mass-density information has allowed characterization of inner-product and outer-product C-S-H gels. The average density of the inner-product C-S-H is smaller than that of the outer product and its variability is larger. Full characterization of the pastes, including segmentation of the different components, is reported and the contents are compared with the results obtained by thermodynamic modelling.

## Abstract 2

The density and content of an amorphous component in cement pastes has been quantified by ptychography in the presence of a large amount of another different amorphous component.

## 1. Introduction   

Portland cement (PC) is considered to be the most manufactured product in the world, currently above 2 × 10^9^ tonnes per year is produced, and it is the main product of the construction industry (Ludwig & Zhang, 2015 ▸). PC is manufactured by adding a setting regulator (a calcium sulfate source) to the Portland clinker and, in many cases, variable amounts of additions and admixtures (Taylor, 1997 ▸). Concrete, made by mixing cement, water, and fine and coarse aggregates, is a composite material with a complex hierarchical microstructure composed of the hydration products of the cement, which glue together the aggregates that act as a granular skeleton. The yearly consumption of concrete is well over 6 km^3^ or more than 2.5 tonnes per person. In spite of its universal use, PC is one of the most environmentally contentious materials since its worldwide production accounts for 6% of the total anthropogenic CO_2_ production (Barcelo *et al.*, 2014 ▸). Hence, there are many ongoing research efforts to better understand cement hydration chemistry in order to reduce its CO_2_ footprint in a safe, economic and sustainable way.

The hydration products of cement depend upon its elemental and mineralogical composition, texture and the hydration conditions: water-to-cement ratio, temperature, pressure, presence of admixtures *etc*. We shall not go into detail about this here but the interested reader is recommended to read the classical books on the chemistry of cements (Bensted & Barnes, 2002 ▸; Taylor, 1997 ▸) and recent books for state-of-the-art characterization techniques (Scrivener *et al.*, 2016 ▸; Pöllmann, 2017 ▸). The main component phases of the hydration of PC are nanocrystalline calcium silicate hydrate (C-S-H) gels, crystalline portlandite, Ca(OH)_2_ and crystalline ettringite, Ca_6_Al_2_(SO_4_)_3_(OH)_12_·26H_2_O. Moreover, Fe–Al siliceous hydrogarnet, the main iron-containing phase, has been reported to form in PC, based on X-ray powder diffraction and X-ray absorption spectroscopy studies (Dilnesa, Wieland *et al.*, 2014 ▸; Vespa *et al.*, 2015 ▸). Its observation and quantification is, in the presence of nanocrystalline C-S-H, difficult and challenging because of its poorly ordered nature.

Furthermore, plain PC can be mixed with additional compounds to decrease the production cost, to lower the CO_2_ footprint and to improve performance. The main addition to plain PC in concrete production is calcite and supplementary cementitious materials (SCM) (Pacewska & Wilińska, 2013 ▸; Scrivener *et al.*, 2015 ▸; Juenger & Siddique, 2015 ▸). SCMs include fly ash (FA), ground granulated blast-furnace slag, calcined clays, natural pozzolans and silica fume.

It is widely accepted that the strength and other mechanical and chemical properties of mortars and concretes mainly rely upon the formation of C-S-H gel during cement hydration which forms about half the volume of all cement hydrates (Taylor, 1997 ▸; Bensted & Barnes, 2002 ▸). Because of the complex hierarchical arrangement of cement pastes in general (Mehta *et al.*, 2014 ▸), and C-S-H gel in particular (Papatzani *et al.*, 2015 ▸; Cuesta *et al.*, 2018 ▸; Andalibi *et al.*, 2018 ▸; Kumar *et al.*, 2017 ▸), techniques yielding spatially resolved information are key to their characterization. There are different approaches for obtaining spatially resolved information including scanning electron microscopy, transmission electron microscopy, nanoindentation and X-ray imaging. Here, we will focus only on synchrotron X-ray imaging techniques (Aranda, 2016 ▸) which allow the investigation of samples or pastes without the need for a preparation step before measurements are made, thus avoiding many artefacts caused by such preparations.

Since the pioneering work of Bentz *et al.* (2000 ▸), absorption-contrast X-ray computed tomography has been widely used for investigating PC hydration and to study several parameters under different conditions, including the pore and void network (Gallucci *et al.*, 2007 ▸; Moradian *et al.*, 2017 ▸), tortuosity (Promentilla *et al.*, 2009 ▸, 2016 ▸), leaching alterations (Sugiyama *et al.*, 2010 ▸), alkali–silica reactions (Marinoni *et al.*, 2012 ▸; Voltolini *et al.*, 2011 ▸; Hernández-Cruz *et al.*, 2016 ▸), early hydration microstructure evolution (Gastaldi *et al.*, 2012 ▸; Parisatto *et al.*, 2015 ▸) and uranium encapsulation in grout (Stitt *et al.*, 2017 ▸, 2018 ▸). As of today, these tomographic investigations have limited spatial resolution (usually 500 nm at best) and it is extremely difficult to distinguish between different hydrated phases as they have very similar X-ray absorption values (Deboodt *et al.*, 2017 ▸) yielding poor contrast. Phase-contrast tomography has also been applied to PC pastes but the final attained spatial resolution was close to 10 µm (Yang *et al.*, 2016 ▸).

One step forward in X-ray computed tomography involves the use of the diffraction signal (Birkbak *et al.*, 2015 ▸). This technique combines the merits of diffraction with computed tomography to allow imaging of the interior of materials, for instance determining the distributions of polymorphs in complex mixtures, by using a focused synchrotron beam and measuring the diffraction or scattering signal arising from the nano- or atomic structure of the specimen in a raster-scanning approach. This technique has also been widely applied to cements (Artioli *et al.*, 2010 ▸; Valentini *et al.*, 2011 ▸, 2012 ▸; Voltolini *et al.*, 2013 ▸), *e.g.* to unravel the change of nucleation mechanism in cements when adding polycarboxyl­ate ether superplasticizers (Artioli *et al.*, 2015 ▸) and to better understand the microstructural signature of carbonation in blended cement pastes (Claret *et al.*, 2018 ▸). Unfortunately, the best spatial resolution attainable today with this technique, ∼4 µm, does not allow the hierarchical arrangement of the component phases in the key 100–5000 nm mesoscale range to be understood.

The state-of-the-art evolution of these synchrotron imaging techniques brings us to X-ray ptychography, a scanning technique that makes use of the coherent properties of synchrotron radiation (Dierolf *et al.*, 2010 ▸). In coherent diffraction imaging, the post-sample X-ray optics are replaced by phase-retrieval algorithms which, combined with the ptychographic approach (scanning a sample at overlapping illumination areas of the sample), makes this technique robust and reliable (Rodenburg *et al.*, 2007 ▸; Guizar-Sicairos & Fienup, 2008 ▸; Thibault *et al.*, 2009 ▸; Faulkner & Rodenburg, 2004 ▸). A focused hard-X-ray synchrotron beam is used to illuminate the sample and coherent diffraction patterns are recorded in the far field. The diversity in the data arising from acquisitions at overlapping areas of the specimen and the use of iterative phase-retrieval algorithms yield both the amplitude and the phase of the complex-valued transmissivity of the sample. X-ray ptychography provides 2D high-resolution projections of the sample. Ptychographic X-ray computed tomography (PXCT) (Dierolf *et al.*, 2010 ▸) combines ptychography and tomography to simultaneously provide two volumes with a 3D distribution of the difference from the real part of the refractive index, δ(**r**), and the imaginary part of the refractive index, β(**r**). Thus, the complete complex-valued refractive index of the sample, *n*(**r**) = 1 − δ(**r**) + *i*β(**r**), can be obtained (da Silva *et al.*, 2015 ▸). We note that this procedure gives two tomograms per measurement with complementary information, volumetric electron density and X-ray attenuation. PXCT can provide isotropic 3D resolution better than 20 nm (Holler *et al.*, 2014 ▸) and accurate volumetric mass density values when the stoichiometries are known (Diaz *et al.*, 2012 ▸), which makes the technique appropriate for analysing the microstructures of cement pastes (Trtik *et al.*, 2013 ▸; da Silva *et al.*, 2015 ▸; Cuesta *et al.*, 2017*a* ▸), as the densities of every component within complex mixtures can be mapped out with a high spatial resolution. Hereafter, the term density is used only when referring to volumetric mass density. Volumetric electron density is abbreviated to just electron density. PXCT has been applied to investigate the hydration, composition, density and microstructure of a PC paste (Trtik *et al.*, 2013 ▸) although the reported mass densities were affected by the resin-impregnation procedure employed. The 3D maps of all the individual phases were segmented and their mass densities were quantitatively determined. Moreover, the densities and water content of the C-S-H gels were also determined (da Silva *et al.*, 2015 ▸) for an alite paste with a high water-to-alite ratio. We have recently used this technique to determine the ettringite and gel volume distributions in the segmented tomograms of calcium sulfoaluminate pastes (Cuesta *et al.*, 2017*a* ▸). In this work, the composition and density of two aluminium hydroxide amorphous gel agglomerates were determined. The *in situ* hydration of a sample of ye’elimite with gypsum at different early stages, between 48 and 63 h, was also followed by PXCT (Cuesta *et al.*, 2017*b* ▸). Finally, we are aware of the use of ptychography with soft X-rays for studying different types of cement pastes (Bae *et al.*, 2015 ▸; Geng, Li *et al.*, 2017 ▸; Geng, Myers *et al.*, 2017 ▸; Geng *et al.*, 2018 ▸). The high spatial resolution of this approach is adequate for studying the arrangement of some components within the pastes but the small field of view precludes the quantification of the different components.

In this work, we have used PXCT to study three cement pastes: neat PC, PC blended with calcite and PC blended with FA. In addition to full segmentation, and so quantitative component analysis, several parameters have been determined, which constitutes a significant advance in the understanding of cement chemistry.

## 2. Materials and methods   

### 2.1. Cement pastes   

Commercial PC from FYM SA (Malaga, Spain), FA Class F supplied by a power station in Lada (Spain) (García-Materé *et al.*, 2013 ▸) and CaCO_3_ (calcite, 99.0%) from Sigma–Aldrich were used for this study. Their chemical and mineralogical analyses are given in the Supporting information. Three pastes were studied in this work. These were prepared by mixing the appropriate cement with water in a two-step approach: cement preparation and a water-mixing procedure. The cement samples were prepared as follows.

(*a*) *Neat PC*. Commercial cement was milled for 40 min in a vibratory ball mill. The PC was milled to ensure that large particles could not block the narrow parts of the capillaries, see Fig. S1(*a*).

(*b*) *PC–CC blend*. A mixture of 80 wt% of PC and 20 wt% of CaCO_3_ was prepared by homogenizing the blend in an agate mortar for 15 min. Previously, PC was milled as indicated above. Calcite was attrition milled with iso­propanol for 10 min, see Fig. S1(*b*).

(*c*) *PC–FA blend*. A mixture of 70 wt% of PC and 30 wt% of FA was prepared with the same procedure described above. The initial particle size distribution of FA is reported in Fig. S1(*c*). Additionally, the blend was milled for 30 min in a vibratory ball mill and was then attrition milled with iso­propanol for 30 min.

The long milling times can increase the temperature and could even lead to partial dehydration of gypsum. This possible change in the sulfates could have an effect on the early stage hydration kinetics but it should not significantly modify the hydration phase assemblage at later stages, *e.g.* after five months as studied here.

The three cement samples were loaded inside tapering quartz capillaries. An ultrasound bath was used to shake the capillaries in order to help the powder to reach the tip. The possible separation of particles in this process cannot be ruled out. However the effect, if it takes place, must be minor given the agreement between the content of the determined component phases and the results from the thermodynamic modelling of the nominal starting composition (see below). The capillary was then filled up with an adequate amount of distilled water and both ends of the capillary were sealed immediately with UV-hardening glue. The pastes within the quartz capillaries were stored at room temperature and investigated at the cSAXS beamline after five months of hydration.

### 2.2. PXCT experiment   

The three cement pastes were measured at the cSAXS beamline at the Swiss Light Source, Paul Scherrer Institute (Villigen, Switzerland) using the instrument already described in a previous work (Holler *et al.*, 2014 ▸). The photon energy of the X-ray beam was 6.2 keV. Diffraction patterns were collected with an EIGER 500K detector placed 7.305 m downstream of the sample satisfying the ptychography sampling conditions (Edo *et al.*, 2013 ▸; da Silva & Menzel, 2015 ▸). For the PC sample, the total acquisition time for all diffraction patterns including the time necessary for sample positioning was 17 h. For the PC–CC and PC–FA blends, the total acquisition times were 22 and 20 h, respectively. Additional experimental details about the measurements can be found in the Supporting information. Some limitations apply to PXCT that can be sample dependent, for example, radiation damage, which is mostly a problem for soft condensed matter and labile samples. Another limitation is the width of the samples to be imaged. Very thick samples do not allow enough X-ray penetration and may lead to a very large phase shift that could be difficult to invert. The sample thickness can also limit the achievable spatial resolution in the 2D projection images due to a limit on the depth of focus. This limitation affects any microscopy method, but in the case of ptychography it can be overcome, as discussed by Tsai *et al.* (2016 ▸). Because of its high resolution and its capability to provide quantitative absorption and phase contrast, PXCT is starting to be used for the characterization of a wide range of materials, from integrated circuits (Holler *et al.*, 2017 ▸) to frozen-hydrated brain tissue (Shahmoradian *et al.*, 2017 ▸).

### 2.3. PXCT data processing and analysis   

The ptychography reconstructions were carried out using a difference-map algorithm (Thibault *et al.*, 2009 ▸) followed by a maximum-likelihood refinement (Thibault & Guizar-Sicairos, 2012 ▸). The pixel size of the reconstructed projections was 38.95 nm. The spatial resolution of the tomograms was determined by Fourier shell correlation (FSC) with a threshold based on the half-bit criterion (Holler *et al.*, 2014 ▸; van Heel & Schatz, 2005 ▸). Further specific details about the tomographic reconstructions (Guizar-Sicairos *et al.*, 2011 ▸) can be found in the Supporting information.

The 3D electron-density distribution, *n*_e_(**r**), can be determined as follows (Diaz *et al.*, 2012 ▸)
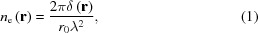
where *r*_0_ is the classical electron radius and λ is the X-ray wavelength. The density can be obtained as
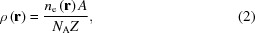
where *N*_A_ is Avogadro’s number, *A* is the molar mass, and *Z* is the total number of electrons in the formula unit.

Moreover, the linear attenuation coefficient, μ, can be calculated by using equation (3) (da Silva *et al.*, 2015 ▸)
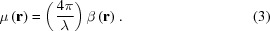



The next stage was detailed spatial characterization of selected component phases. Many component particles were chosen and the spatial distribution of their electron densities was thoroughly studied. The analysis was performed by monitoring the evolution of the electron-density value along selected directions. This characterization was carried out with *ImageJ/Fuji* shareware (Abràmoff *et al.*, 2004 ▸; Schindelin *et al.*, 2012 ▸).

Following this, the segmentation of the component phases was carried out. A region of interest of each sample was selected to perform threshold-based image segmentation initially on the phase-contrast tomogram. The segmentation study was performed with *Avizo Fire* v. 8.0 (FEI Visualization Sciences Group). All the materials were separated using the average values obtained for the electron densities by applying the threshold tool which is included in the segmentation editor of the *Avizo* suite. The borders of the regions were smoothed. Finally, the volume percentages of each phase were quantitatively determined using the material statistics tool of the *Avizo* suite. Moreover, the average electron-density values for every component were also obtained from these segmented volumes.

The quality [signal-to-noise (s/n) ratio] and spatial resolution of the absorption tomograms were poorer than those of the phase-contrast tomograms. This is an inherent feature in the transmissivity of a sample with hard X-rays, *i.e.* X-rays with an energy above about 2 keV, where absorption is much weaker compared with the phase shift. Consequently, the 3D segmented masks of the material phases based on the phase-contrast tomograms were used in the amplitude tomograms to obtain the attenuation coefficient based on the β values for the main mineralogical phases. In addition, the *Shrink* tool was applied for these tomograms. This tool applies morphological erosion of the mask using a structural element that includes the voxel of origin and its connection to neighbouring voxels.

Moreover, and very importantly, for phases with very similar electron densities, because of the difficulty of distinguishing them in the phase-contrast tomogram, the β(**r**) dataset was also used to perform the segmentation procedure. In these cases, the segmented masks were created by providing the lower and upper bounds for both δ and β values obtained from the bivariate histogram (see below).

### 2.4. Thermodynamic modelling   

Thermodynamic modelling was carried out using Gibbs free energy minimization software (*GEMs* 3.4; Wagner *et al.*, 2012 ▸; Kulik *et al.*, 2013 ▸) which calculates the equilibrium phase assemblages in chemical systems from their total bulk elemental composition. The default databases were expanded with the CEMDATA18 database (Lothenbach *et al.*, 2019 ▸); C-S-H was modelled with the CSH-II model, and the Parrot and Killoh model was used for the hydration modelling (Lothenbach *et al.*, 2008 ▸). The CSH-II model was selected because it has 2.1 H_2_O/Si for C-S-H without gel water and a high Ca/Si ratio for the mineral phase, which results in adequate overall water content, close to 4.0 water molecules per mol of Si which includes gel water, and density values in agreement with the measurements of Muller *et al.* (2013 ▸). Conversely, the water content for C-S-H without gel water in the more recent CSHQ model is 3 H_2_O/Si, which is too high for the studied samples (Lothenbach *et al.*, 2019 ▸). The reaction of the amorphous part of the FA was modelled using the kinetic model outlined by De Weerdt *et al.* (2011 ▸).

### 2.5. Relevance of the spatial resolution   

On the one hand, X-ray diffraction computed tomography is used for studying cement pastes with a resolution ranging from 4 to 10 µm (Artioli *et al.*, 2015 ▸; Claret *et al.*, 2018 ▸). Although this spatial resolution is adequate for studying large particles (unreacted calcium silicates, and some phases such as portlandite and calcium carbonate), it is not suitable for unravelling the complex hierarchical arrangement of component phases within the pastes with particle sizes ranging from 0.1 to 5 µm. On the other hand, absorption-contrast computed tomography may acquire tomograms with a spatial resolution of 0.5 µm (Zhang, 2017 ▸) but the absence of proper contrast between the different hydrated component phases makes this approach of little use for distinguishing hydrates, although it is relevant for the analysis of porosity and other features with high X-ray absorption (aggregates, metal bars *etc*.). The different hydrated component phases in cement phases have particle sizes of the order of several micrometres (or smaller) and so a spatial resolution of about 100 nm with sufficient contrast is vital to minimize the partial volume effect in the segmentation step.

Finally, even if the voxel size is ∼40 nm, the actual 3D spatial resolution can be limited by other factors such as the already mentioned contrast between different components in the sample. In this work, the actual 3D spatial resolution ranges between 56 and 80 nm for the phase contrast (δ) tomograms and it is about 250 nm for the absorption (β) tomograms.

### 2.6. C-S-H chemical composition at different length scales   

C-S-H gel composition and derived properties such as volumetric mass density may vary depending upon a number of factors including the preparation conditions (Jennings, 2008 ▸; Roosz *et al.*, 2016 ▸). The interested reader is directed towards recent reviews on C-S-H for detailed information (Richardson, 2008 ▸; Jennings, 2008 ▸; Papatzani *et al.*, 2015 ▸; Palkovic *et al.*, 2016 ▸). At the nanometre scale, it has been shown recently that C-S-H from hydrating alite had a defective clinotobermorite structure, with an approximate composition of Ca_11_Si_9_O_28_(OH)_2_·8.5H_2_O, and monolayers of Ca(OH)_2_ and gel water (Cuesta *et al.*, 2018 ▸). Nanocrystalline C-S-H, particle size ≃ 5 nm, has a Ca/Si molar ratio of ∼1.3; which has been previously reported (Cong & Kirkpatrick, 1996 ▸; Skinner *et al.*, 2010 ▸; Chen *et al.*, 2010 ▸; Richardson, 2014 ▸; Grangeon *et al.*, 2017 ▸; Cuesta, Zea-Garcia *et al.*, 2017 ▸; Andalibi *et al.*, 2018 ▸)

However, it is very well established that the neat PC pastes produce C-S-H gels with an average Ca/Si molar ratio of 1.7–1.8 at the micrometre scale (Bensted & Barnes, 2002 ▸; Taylor, 1997 ▸) with an average stoichiometry close to (CaO)_1.8_SiO_2_·4H_2_O. There is still some debate if the excess of calcium with respect to the defective clinotobermorite structure, Ca/Si ratio ≃ 1.3, is caused by the presence of monolayers of Ca(OH)_2_ (Grangeon *et al.*, 2017 ▸; Cuesta *et al.*, 2018 ▸) or by the layers of calcium hydroxide intergrown within the clinotobermorite nanoparticles (Kumar *et al.*, 2017 ▸). In each case, the component phase in the cement pastes with a Ca/Si molar ratio ≃ 1.8 at the micrometre scale originates from the fine intermixing at the nanometre scale of defective tobermorite and calcium hydroxide.

Finally, it is noted that blended PC pastes with SCM tend to have C-S-H gels with lower Ca/Si ratios at the micrometre scale ranging between 1.4 and 1.7 (Lothenbach *et al.*, 2011 ▸; Deschner *et al.*, 2012 ▸).

### 2.7. Relevance of the combined use of δ and β datasets   

Acknowledging the intrinsic lower spatial resolution in the reconstructed absorption tomograms, here we highlight the importance of having this complementary information. For instance, MgO and Ca_4_Al_2_Fe_2_O_10_ have electron-density values of 1.07 and 1.10 e Å^−3^, respectively. This 3% contrast in the δ-tomograms makes the independent segmentation of these components virtually impossible. However, MgO and Ca_4_Al_2_Fe_2_O_10_ have attenuation coefficient values of 217 and 566 cm^−1^, respectively. This 60% contrast in the β-tomograms enables the independent segmentation of these components. In fact, the software used allows one to carry out a simultaneous segmentation of the δ- and β-tomograms which allows one to profit from the improved spatial resolution in the phase-contrast dataset and the additional attenuation contrast in the absorption datasets.

The example given above is not unique. The C-S-H gel and crystalline Ca(OH)_2_ phases also have similar electron-density values of ∼0.66 and 0.69 e Å^−3^, respectively, but their attenuation coefficients of ∼280 and 446 cm^−1^ provide a high absorption contrast.

### 2.8. Particle size distribution   

The average particle size and the particle size distribution for the samples were measured using laser diffraction employing an analyser (MastersizerS, Malvern, UK) with a wet sample cell (using ethanol as an organic medium).

## 3. Results and discussion   

### 3.1. Nomenclature for the component phases   

In this work, 11 component phases are described. For an adequate understanding, Table 1 ▸ shows the chemical formula of the phases, in which an approximation sign is given for the nanocrystalline/amorphous components, and the corresponding numerical labels used in the figures and the abbreviations used in the text.

### 3.2. Water-to-solid (w/s) ratios   

The estimation of this ratio is crucial for describing and understanding the hydration behaviour of cement pastes. First, the component phase assemblage of a neat PC paste was investigated by PXCT. The volume of the reconstructed dataset for this sample was about 4.8 × 10^4^ (40 × 40 × 30) µm^3^. The nominal w/s used ratio was 1.0 but, as reported previously (Gallucci *et al.*, 2007 ▸; Parisatto *et al.*, 2015 ▸; Cuesta *et al.*, 2017*a* ▸), it is hard to control the w/s ratio homogeneity along the full length of very narrow capillaries (diameters ranging from 30 to 100 µm). However, it is possible to estimate the w/s ratio of the scanned capillary region from the final measured average attenuation coefficient, 348.5 cm^−1^ (excluding 2.2 vol% of air porosity), see Table 2 ▸. The elemental (Table S1) and mineralogical compositions (Table S2 and Fig. S2) of the used PC are given in the Supporting information. From these analyses, the average μ of the anhydrous PC was 624.9 cm^−1^. The μ value of free water is 22.2 cm^−1^. Hence, it can be estimated that the paste was composed of 54.0 vol% PC and 46.0 vol% water to justify the overall μ of the paste. This calculation is approximate as it neglects the possible effect of the shrinkage, but this error must be smaller than 8%. This simple calculation yields a w/s [in this case it is the same as the water-to-cement (w/c) mass ratio] of 0.27, which is equivalent to a volume ratio of 0.85. This w/s ratio value is totally consistent with no capillary pore solution and 20 vol% of unreacted PC phases, see below.

Then, two other pastes were investigated: PC–CC and PC–FA blends. PC–CC blend contained 20 wt% (or 22.2 vol%) of calcite and PC–FA blend contained 30 wt% (or 33.5 vol%) of FA. Calcite sample contains 100 wt% of CaCO_3_ (see Fig. S3). The elemental (Table S3) and mineralogical compositions (Table S4 and Fig. S4) of the FA sample are also given in the Supporting information. The nominal w/s ratio was 1.0 for both cases. On the one hand, the dry PC–CC blend had an average μ value of 581.5 cm^−1^. So, considering the μ value of water, excluding air porosity and neglecting the shrinkage, this paste, with an average μ value of 341.2 cm^−1^, is estimated to be composed of 56.0 vol% of blended PC–CC cement and 44.0 vol% water. So, the w/s mass ratio is estimated to be 0.27 (which is equivalent to a w/c mass ratio of 0.33, and w/s volume ratio of 0.79 or w/c volume ratio of 0.99). On the other hand, the PC–FA blend had an average μ value of 479.3 cm^−1^. Again considering the μ value of water, excluding air porosity and neglecting the shrinkage, this paste, with average μ value of 267.3 cm^−1^, is estimated to be composed of 53.6 vol% of blended PC–FA cement and 46.4 vol% water. Hence, the w/s mass ratio is estimated to be 0.30 (which is equivalent to a w/c mass ratio of 0.43, and w/s volume ratio of 0.87 or w/c volume ratio of 1.31). These values are summarized in Table 2 ▸.

### 3.3. PXCT electron densities and attenuation coefficients   

PXCT yields two tomographic datasets: the 3D electron-density distribution, *n*_e_(**r**), obtained from the phase projections, and the 3D distribution of the complex part of the refraction index, β(**r**), obtained from the absorption projections. As expected, the resolution in the *n*_e_(**r**) dataset is better in terms of noise and resolution than that in the β(**r**) one. The 3D spatial resolutions for the *n*_e_(**r**) datasets, determined by FSC, were estimated to be 80, 56 and 59 nm (Fig. S5), for neat PC, PC–CC and PC–FA blends, respectively. The 3D spatial resolutions for the β(**r**) datasets were estimated to be around 250 nm.

#### 3.3.1. Neat PC paste   

**Figure 1:**
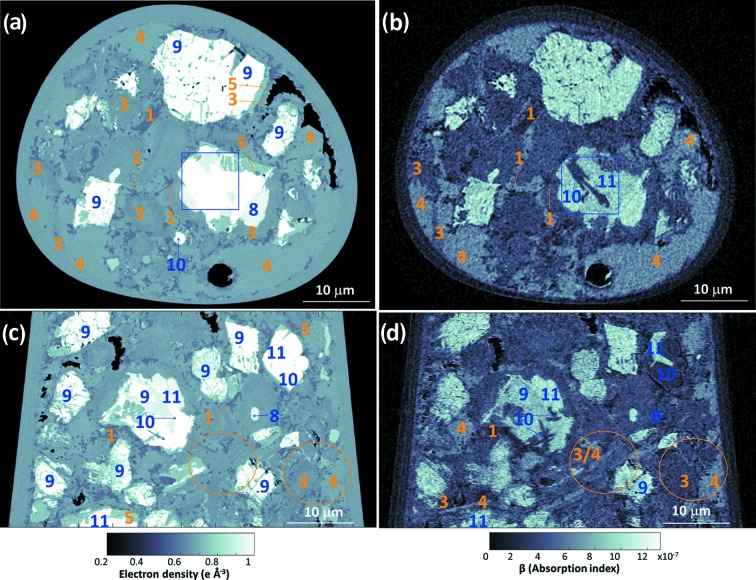
Selected slices of the PXCT tomograms for neat PC paste after five months of hydration at room temperature. (*a*) A horizontal slice of the electron-density dataset, (*b*) the corresponding slice of the absorption dataset, (*c*) a vertical slice of the electron-density dataset and (*d*) the corresponding slice of the absorption dataset. Some regions are identified as different component phases, based on the electron-density values, using the labelling system shown in Table 1 ▸. The highlighted regions with the HD_C-S-H/portlandite (phases 3/4 in orange circles) pair and MgO/C_4_AF (phases 10/11 in blue squares) pair are discussed in the text.

Selected horizontal and vertical slices for the *n*_e_(**r**) tomogram are shown in Figs. 1 ▸(*a*) and 1 ▸(*c*), respectively. The corresponding slices in the β(**r**) tomogram are shown in Figs. 1 ▸(*b*) and 1 ▸(*d*), respectively. Eight different component phases were identified in the *n*_e_(**r**) tomogram based on their electron densities (grey levels).

The qualitative analysis of Fig. 1 ▸ already gives valuable information. The air porosity content (black regions within the capillary) is very small. The grey level in the *n*_e_(**r**) tomogram is lighter as the electron density for different phases increases. AFt and C_4_AF being the components with lowest and highest electron densities, respectively. As discussed above, the electron densities of C_4_AF and MgO are very close and they cannot be distinguished in Figs. 1 ▸(*a*) and 1 ▸(*c*). However, as shown in the blue square in Figs. 1 ▸(*a*) and 1 ▸(*b*), these components can be easily differentiated in the (slightly noisier) absorption tomogram. More importantly, C-S-H gel and portlandite also have quite close electron-density values, so they can hardly be discriminated in the electron-density tomogram. However, these components can be readily distinguished in the absorption tomogram, see the brown circles in Figs. 1 ▸(*c*) and 1 ▸(*d*). PXCT tomograms revealed different shapes for portlandite volumes as shown previously by electron microscopy. They range from irregular forms with sizes well above 15 µm to quite thin plaques (thicknesses smaller than 0.5 µm) interspersed between C-S-H volumes, see Fig. 1 ▸(*d*).

Ten random regions of particles were analysed for each component phase to determine the electron densities, which were then converted to mass densities by using equation (2) and are given in Table 3 ▸. In general, the water content of C-S-H is variable, hence the water content was determined as explained below, and then the electron density could be converted to volumetric mass density. It can be observed that there is a very good agreement between both measured and theoretical mass densities for crystalline component phases (where the theoretical mass densities can be well defined). The average relative error is lower than 1.5%. The electron densities have also been obtained from the segmentation volumes using *Avizo* software. A small systematic variation between the measured and theoretical attenuation coefficients has been observed in the three pastes. The origin of this disagreement is not clear to us. However, as there are several phases with well known attenuation coefficients (for instance, the capillary, calcite and portlandite), we have used these values to calculate the correction parameter which was 1.05 (*i.e.* 5%). Hence, all reported attenuation coefficients in this work (for the three pastes) are determined from the complex part or the refraction index datasets multiplied by the correction factor 1.05.

**Figure 2:**
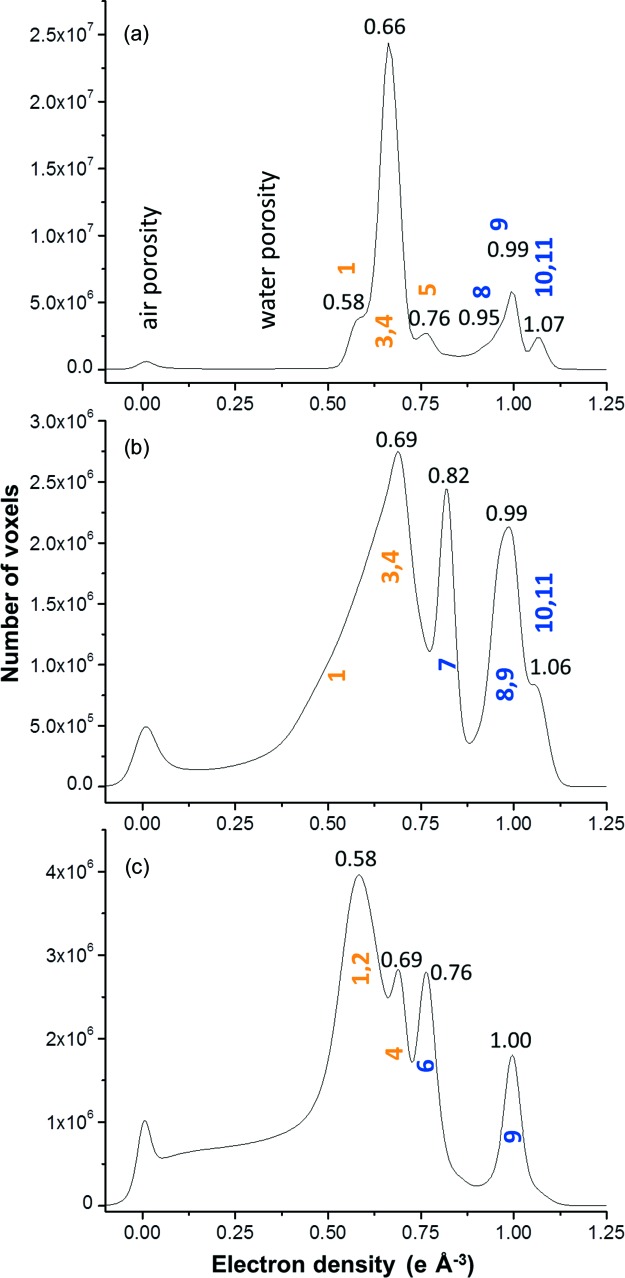
VOI histograms of the electron densities for (*a*) neat PC paste, (*b*) PC–CC blend paste and (*c*) PC–FA blend paste, after five months of hydration. Air and water porosity regions are indicated. Corresponding component phases are assigned to the different peaks using the labelling system shown in Table 1 ▸.

An overall picture of the components can be obtained from the electron-density histogram of a volume-of-interest (VOI) inside the capillary of about 1.6 × 10^4^ µm^3^, see Fig. 2 ▸(*a*). The shift in the air peak is caused by partial volume effects as the computed region may contain liquid/solid phases below the resolution of the data. The small peak at *n*_e_ = 0.58 e Å^−3^ corresponds to AFt, see Fig. 2 ▸(*a*) and Table 3 ▸. Fig. 2 ▸(*a*) displays the strongest peak at *n*_e_ ≃ 0.66 e Å^−3^. As it is shown in Table 3 ▸ that peak is caused by two phases, one having *n*_e_ ≃ 0.65 e Å^−3^ (C-S-H gel) and another with *n*_e_ = 0.69 e Å^−3^ (CH). Furthermore, Fig. 2 ▸(*a*) displays a small peak at *n*_e_ ≃ 0.76 e Å^−3^ (see also Table 3 ▸). It will be shown below that this peak corresponds with Fe–Al siliceous **h**ydro**g**arnet (Fe–Al–Si–**Hg**). The remaining anhydrous cement phases have electron densities larger than 0.94 e Å^−3^, see Fig. 2 ▸(*a*) and Table 3 ▸. The density determination requires knowledge of the composition, so it is straightforward for crystalline phases, but some assumptions need to be made for phases with variable stoichiometry, developed below, see Table 3 ▸.

**Figure 3:**
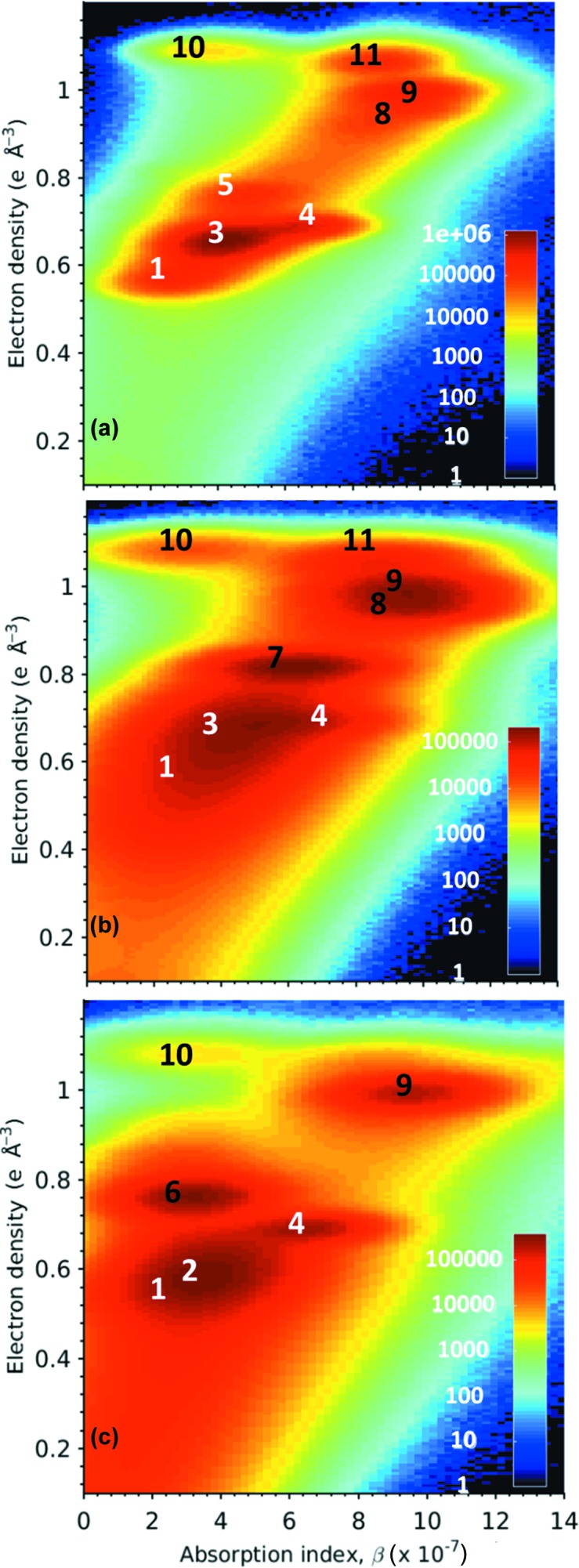
Bivariate histograms of absorption indexes (β) and electron densities for (*a*) neat PC paste, (*b*) PC–CC blend and (*c*) PC–FA blend, after five months of hydration. Corresponding component phases are assigned to the different peaks according to the labelling system given in Table 1 ▸.

Fig. 3 ▸(*a*) shows the 2D bivariate plot where the number of voxels is plotted as a function of both δ and β values, where the assignment of the different peaks to the corresponding component phase is also given. In this plot, some component phases with very similar electron densities are evident (*y* values) but in those cases they exhibit different attenuation coefficients (*x* values). Thus, the main peak at *n*_e_ ≃ 0.66 e Å^−3^ [Fig. 2 ▸(*a*)] has two contributions, from C-S-H (phase 3) and portlandite (phase 4), clearly shown in the bivariate histogram. Furthermore, the small peak at *n*_e_ ≃ 1.07 e Å^−3^ [Fig. 2 ▸(*a*)] has two contributions, from MgO (phase 10) and C_4_AF (phase 11) as noticeable in Fig. 3 ▸(*a*).

#### 3.3.2. PC–CC blend paste   

**Figure 4:**
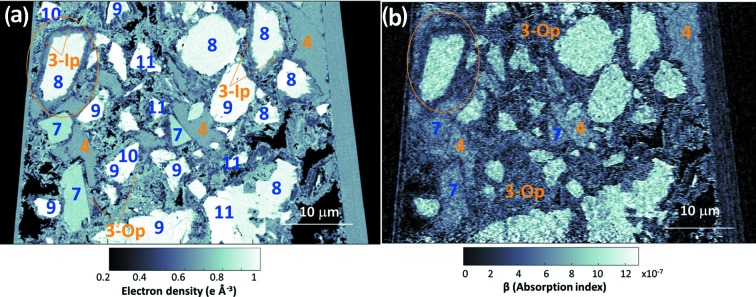
Selected slices of the PXCT tomograms for PC–CC blend paste after five months of hydration at room temperature. (*a*) A vertical slice of the electron-density dataset and (*b*) the same slice of the absorption dataset. Some regions are identified as different component phases based on the electron-density values, using the labelling system given in Table 1 ▸. 3-Ip refers to phase 3 (C-S-H gel) with the inner-product morphology. 3-Op refers to phase 3 (C-S-H gel) with the outer-product morphology.

The reconstructed dataset was about 6 × 10^4^ (45 × 45 × 30) µm^3^. Figs. 4 ▸(*a*) and 4 ▸(*b*) display vertical slices for the *n*_e_(**r**) and β(**r**) tomograms, respectively. It can be noted that the s/n ratio of the absorption tomogram for this sample is slightly lower than that of the neat PC pastes [Fig. 1 ▸(*d*)]. The air porosity content for this sample is also larger, see black regions in the bottom part of Fig. 4 ▸ and also the peak in Fig. 2 ▸(*b*). Moreover, the added component (calcite, phase 7) is easily identifiable by its grey value and the straight edges of the particles.

The analysis of the electron-density tomogram revealed the presence of several partially reacted C_3_S particles. This is highlighted in Fig. 4 ▸ and it allows one to investigate the electron/mass densities of the inner product C-S-H gel (labelled 3-Ip) and outer product C-S-H gel (labelled 3-Op). We recall that it is well known in the cement field that C-S-H gel can grow in the volume formerly occupied by the C_3_S particle and then is called inner product, or in the water pore or on other surfaces like calcite, and in this case is termed outer product (Diamond, 2004 ▸; Soin *et al.*, 2013 ▸; Chen *et al.*, 2010 ▸). These two types of C-S-H gels (morphologically different) are highlighted in Fig. 4 ▸. A thorough study of the density of C-S-H gel is discussed below, in the spatial characterization section[Sec sec3.3].

Table 4 ▸ reports the electron and mass densities and the attenuation coefficient values determined from ten random regions of each component phase. An overall picture of the components can be obtained from the electron-density histogram of a VOI inside the capillary, 1.6 × 10^4^ µm^3^, see Fig. 2 ▸(*b*). The added calcite is evident as a strong peak at *n*_e_ = 0.82 e Å^−3^. It is also readily observable that the fraction of unreacted cement components, those having electron densities larger than 0.94 e Å^−3^, is larger than in the neat PC sample, see Fig. 2 ▸(*a*). The strongest peak at *n*_e_ ≃ 0.69 e Å^−3^ is very broad, and the bivariate plot, see Fig. 3 ▸(*b*), clearly shows that it is composed of C-S-H gel and CH. Moreover, the shoulder of this peak towards lower electron-density values is indicative of the presence of AFt and even phases with lower density values, likely to be dispersed calcium aluminate mono­sulfate type phases including monocarboaluminate, AFm (Matschei *et al.*, 2007*a* ▸,*b* ▸; Baquerizo *et al.*, 2015 ▸).

#### 3.3.3. PC–FA blend paste   

**Figure 5:**
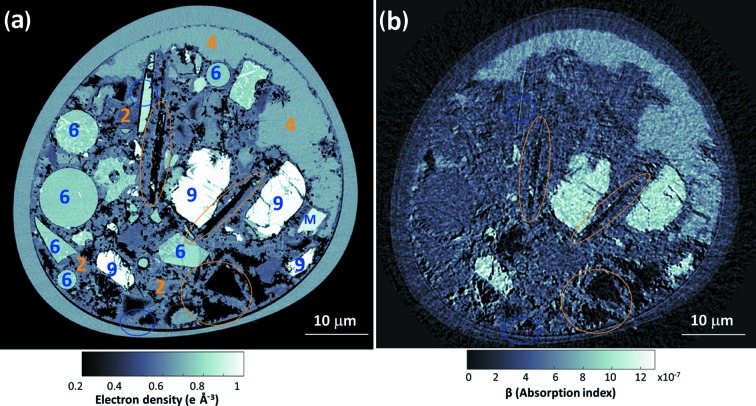
Selected slices of the PXCT tomograms for PC–FA blend paste after five months of hydration at room temperature. (*a*) Horizontal slice of the electron-density dataset and (*b*) same slice of the absorption dataset. Some regions are identified as different component phases, based on the electron-density values, using the labelling system in Table 1 ▸. Air porosity that is likely to be caused by portlandite dissolution is highlighted in pale brown. Tiny empty spaces, that are likely to be caused by chemical shrinkage, are highlighted in blue.

The reconstructed dataset for this sample was 6 × 10^4^ (45 × 45 × 30) µm^3^. Figs. 5 ▸(*a*) and 5 ▸(*b*) display horizontal slices for the *n*_e_(**r**) and β(**r**) tomograms, respectively. The air porosity content for this sample is also large, see black regions in Fig. 5 ▸ and also the peak in Fig. 2 ▸(*c*). Moreover, the added phase (mainly SiO_2_ from the FA, phase 6) is easily identifiable by its grey value and the spherical shape of many particles.

There are three features readily observable in Fig. 5 ▸ that should be discussed. Firstly, there are many unreacted FA particles, this is also evident in Fig. 2 ▸(*c*) (peak at *n*_e_ ≃ 0.76 e Å^−3^) and in Fig. 3 ▸(*c*) (peak assigned to phase 6). Secondly and importantly, there is air porosity with straight edges highlighted in brown in Fig. 5 ▸. Air porosity formed in the mixing stage is expected to have spherical (or somewhat irregular) shape but not plaque shape with straight edges. We are forced to conclude that this isolated air porosity arose from the dissolution of portlandite in the hardened state and there was not enough liquid phase to fill these pores. The dissolution of portlandite is expected from the pozzolanic reaction where the reactive SiO_2_ component of the FA reacts with portlandite to give additional C-S-H at later hydration ages (Papadakis, 1999 ▸; Hanehara *et al.*, 2001 ▸). Thirdly, there are empty spaces very likely to be caused by shrinking, in Fig. 5 ▸, which have been highlighted in blue. This shrinkage, which was not shown for neat PC and PC–CC pastes, is developed probably because of its large w/c mass ratio which was estimated to be ∼0.43, larger than those of the other pastes, ∼0.27–0.33, see above. Furthermore, this larger w/c ratio led to the full consumption of C_3_S and C_4_AF component phases, see Fig. 3 ▸(*c*), while only low-reactive cement phases (C_2_S and MgO) remained.

The most conspicuous feature observable in Fig. 2 ▸(*c*), in the electron-density histogram within a volume of about 2.0 × 10^4^ µm^3^, in addition to the presence of FA (peak at *n*_e_ ≃ 0.76 e Å^−3^), is that the electron-density peak of the main component C-S-H gel is situated at *n*_e_ ≃ 0.58 e Å^−3^. This value is much smaller than those of neat PC and PC–CC pastes (*n*_e_ ≃ 0.66–0.69 e Å^−3^), directly indicating that this C-S-H gel has lower density than those of the previous pastes. This is totally in agreement with a larger w/c ratio estimated from the overall absorption and the full hydration of C_4_AF and C_3_S component phases. Finally, the small peak at pastes *n*_e_ = 0.69 e Å^−3^ is caused by portlandite, as expected.

Table 5 ▸ reports the electron and mass densities and the attenuation coefficient values determined from ten random regions of each component phase. It must be highlighted that this was the only paste where regions of water capillary porosity were observed, *n*_e_ ≃ 0.33 e Å^−3^. The regions of C-S-H gel also contain ettringite and they could not be disentangled as both electron density (*n*_e_ ≃ 0.57–0.59 e Å^−3^) and attenuation coefficient (μ ≃ 190–230 cm^−1^) values are too close.

### 3.4. Spatial characterization of selected component phases   

A thorough analysis of the spatial distribution of the electron density for all component phases, hydrates and partly reacted cement components, is out of the scope of this paper and it will be the subject of a subsequent work. Here we focus on key observations with implication for the nanocrystalline component phase determination. This is the outstanding contribution of PXCT to the cement hydration chemistry.

**Figure 6:**
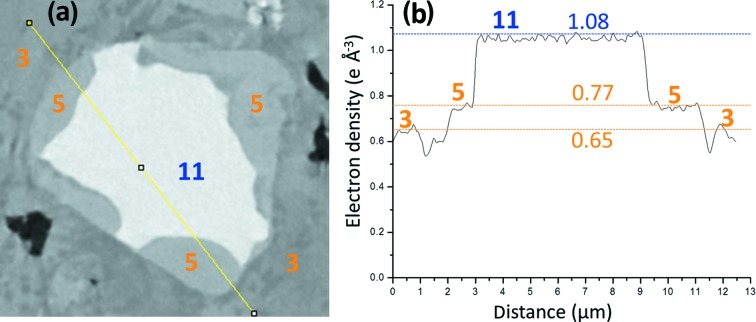
(*a*) A partially reacted C_4_AF particle surrounded by hydrated component phases from the electron-density tomogram of the neat PC paste. The different phases and a line to show the electron-density values are also shown. (*b*) Electron-density values corresponding to the yellow line in (*a*), with horizontal lines showing the average values of the electron densities obtained for the component phases using ten different particles, data from Table 3 ▸. It clearly shows, as an example, how phase 5 encloses the unreacted fraction of the C_4_AF particle. From the electron-density value and its spatial arrangement, phase 5 is concluded to be Fe–Al siliceous hydrogarnet.

Firstly, Fig. 6 ▸(*a*) displays a partially reacted C_4_AF particle surrounded by hydrated component phases from the neat PC paste. The composition of the unreacted particle was identified because of its electron-density value, see Fig. 6 ▸(*b*). The horizontal lines correspond with the average values of the electron densities obtained for the component phases using ten different particles as reported in Tables 3 ▸ to 5 ▸. Very importantly, C_4_AF particles were almost invariably surrounded by a component phase with electron-density value close to 0.77 e Å^−3^. This component phase gives rise to a small peak in Figs. 2 ▸(*a*) and 3 ▸(*a*), as already discussed. From its spatial arrangement and its electron-density value, we conclude that component phase 5 is Fe–Al siliceous hydrogarnet. This spatial arrangement has been already described by electron microscopy, see Fig. 8 of the work by Dilnesa, Wieland *et al.* (2014) ▸. But here we can estimate its density in an untreated sample.

**Figure 7:**
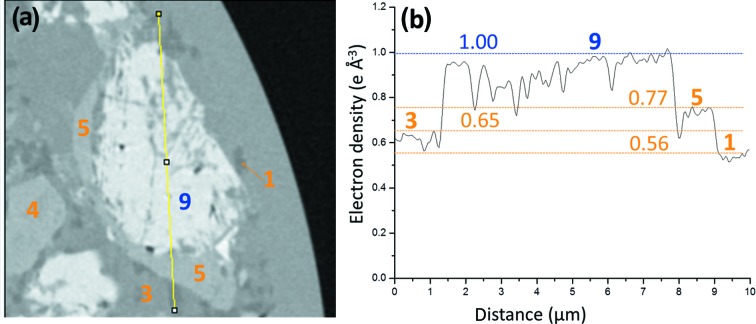
(*a*) A partially reacted C_2_S particle within hydrated component phases from the electron-density tomogram of the neat PC paste. The component phases and a line to show the variation of the electron-density values are also displayed. (*b*) Electron-density values corresponding to the highlighted line are shown together with the horizontal lines described in Fig. 6 ▸.

Secondly, a selected C_2_S particle from the neat PC paste is shown in Fig. 7 ▸(*a*) which was identified by its electron-density value, see Fig. 7 ▸(*b*), and also because of its characteristic pattern of internal defects. This region has been selected for three main reasons. (i) It displays component phase 5, Fe-Al-Si-**Hg**, not directly associated to C_4_AF. This behaviour is not common but it has been observed in some regions. (ii) The region contains all the hydrated component phases observed in this paste, and shown here at higher resolution. (iii) The electron-density pattern within belite shows that hydration takes place along the defects, if they are connected to the particle surfaces.

**Figure 8:**
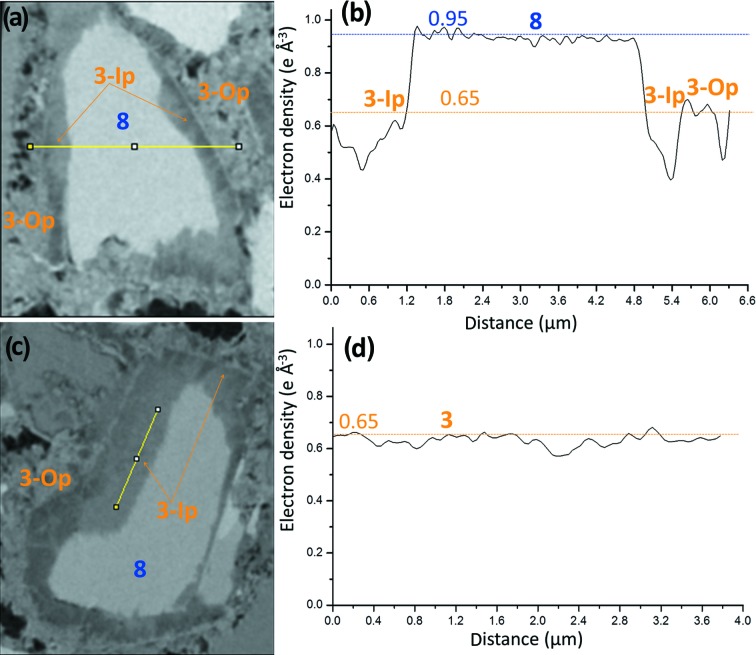
Partially reacted C_3_S particles [plots (*a*) and (*c*)] within hydrated component phases from the electron-density tomogram of the PC–CC blend paste. The component phases and the lines to show the variation of the electron-density values are also displayed. Electron-density values corresponding to the highlighted lines in panels (*a*) and (*c*) are shown in the panels (*b*) and (*d*), respectively. Horizontal lines show the average values of the electron densities obtained for the component phases using ten different particles, data from Table 4 ▸.

Thirdly, two selected C_3_S particles from the PC–CC blend paste are shown in Figs. 8 ▸(*a*) and 8 ▸(*c*) and were identified by its electron-density value, see Fig. 8 ▸(*b*). These two particles, as examples, have been chosen to highlight the electron-density differences between Ip and Op C-S-H gels. Furthermore, the large density variation within Ip regions is also shown in Figs. 8 ▸(*a*) and 8 ▸(*b*). In that figure, it is evident that there are regions in Ip C-S-H gel with electron-density values significantly lower than 0.65 e Å^−3^. It is also apparent from Fig. 8 ▸(*a*) that Ip C-S-H gel has filiform denser structures connecting the unreacted C_3_S core with the outer space. Although Ip in this paste is rather heterogeneous in density, Fig. 8 ▸(*c*) has been selected to show a region of Ip C-S-H gel where this heterogeneity is not large. Ip C-S-H is firmly established as it surrounds unhydrated alite particles. However, Op C-S-H is always a choice based on the surrounding environment but we cannot ensure the absence of a fully hydrated alite particle in that volume. Our direct observations support the very recent needle model proposed for the growth of C-S-H from alite (Ouzia & Scrivener, 2019 ▸), also applicable to Ip C-S-H and not only to Op C-S-H. The density variation within Ip C-S-H is commonly larger than that of Op C-S-H. Fig. S6 displays two selected regions in the electron-density tomogram of the PC–CC blend paste highlighting the electron-density variations in Op C-S-H gel, to be compared with the variations observed in Fig. 8 ▸ for Ip C-S-H gel.

**Figure 9:**
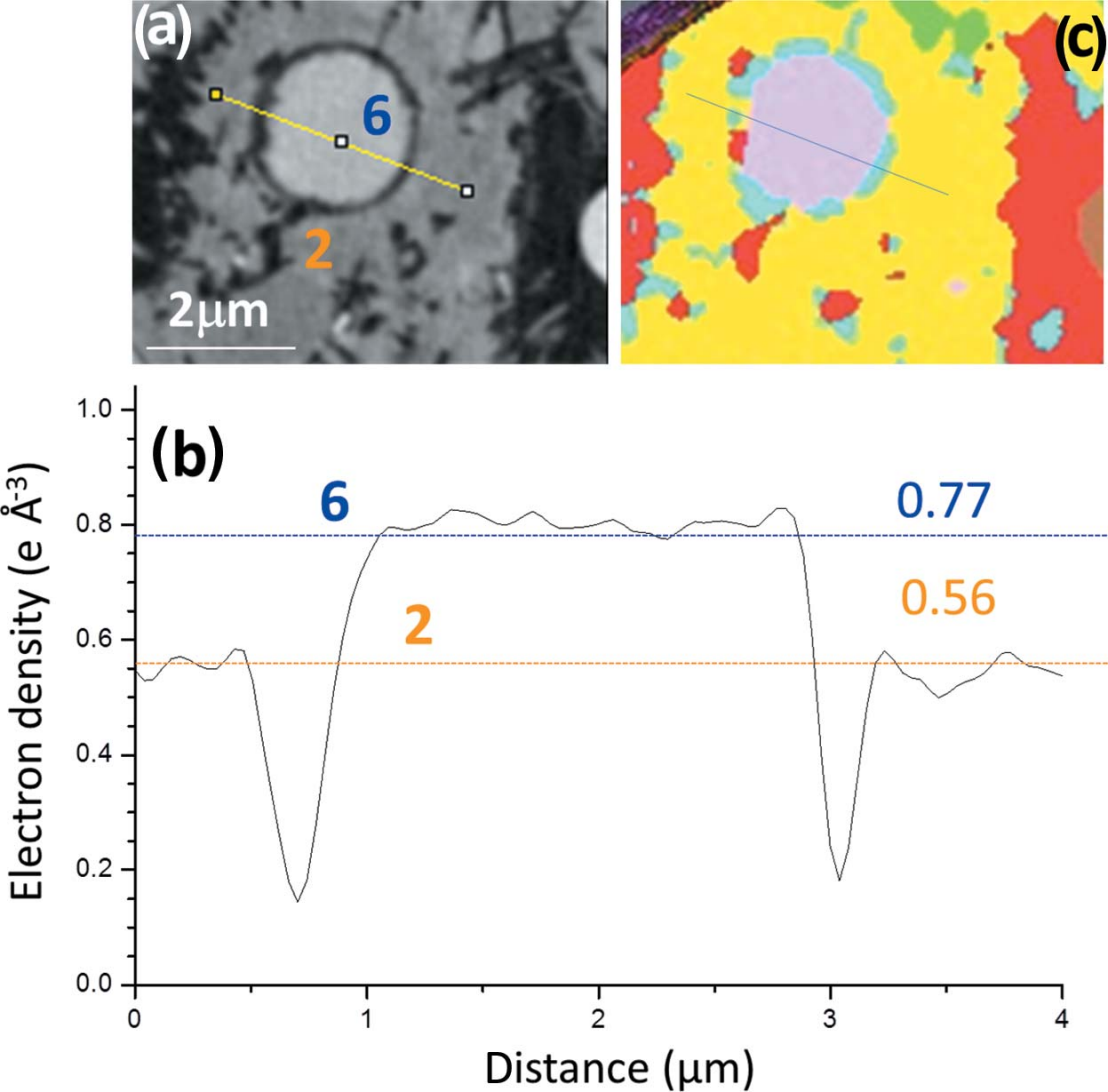
(*a*) Unreacted spherical FA particle within hydrated component phases from the electron-density tomogram of the PC–FA blend paste. The component phases and a line to show the variation of the electron density values are also displayed. (*b*) Electron-density values corresponding to the highlighted line are also shown together with horizontal lines showing the average values of the electron densities obtained for the component phases using ten different particles, data from Table 5 ▸. (*c*) Segmented volumes of the region shown in panel (*a*). Colour codes: red, air porosity; light blue, water porosity; yellow, C-S-H gel (and AFt); light green, CH; light pink, FA; and brown, C_2_S.

Finally, one unreacted FA spherical microparticle (diameter smaller than 2 µm) from the PC–FA blend paste is shown in Fig. 9 ▸(*a*) and was identified by its electron-density value and its very characteristic shape, see Fig. 9 ▸(*b*). This region has been selected for three main reasons. (i) To show the relatively small variability of the electron densities of the two amorphous components. (ii) To show the typical shrinking surrounding the unreacted FA particles, (empty/black) region enclosing the spherical particles. (iii) To visually highlight the good spatial resolution of the images as features slightly smaller than 200 nm are clearly evident. On the other hand, the electron density at the empty region falls sharply, see Fig. 9 ▸(*b*), but the gap is not fully resolved, *i.e.* the electron densities do not fall to zero. The origin of some cracks because of water loss along the hydration process caused by defective sealing cannot be ruled out. Finally, Fig. S7 displays a selected region in its electron-density tomogram highlighting the electron-density variations in the low density C-S-H gel.

### 3.5. Density characterization of the nanocrystalline/amorphous phases   

The chemical composition of the C-S-H gel must be known to determine the density from the PXCT data. For this purpose, the water content must be estimated from the neat PC paste PXCT data, using the β and *n*_e_ values, as previously reported (da Silva *et al.*, 2015 ▸; Cuesta *et al.*, 2017*a* ▸) and detailed in the Supporting information. For estimating the water content, the Ca/Si ratio within the C-S-H gel must be known. We have assumed an overall Ca/Si ratio of 1.80 (Cuesta *et al.*, 2018 ▸) which is in agreement with previous studies (Richardson, 2008 ▸; Papatzani *et al.*, 2015 ▸). Following this methodology, the water stoichiometry determined for the gel was (CaO)_1.80_SiO_2_(H_2_O)_3.96_ and then a density of 2.11 g cm^−3^ is obtained. It is underlined that the Ca/Si ratio plays a larger role for the water-content determination than for the density result. Thus, if an average Ca/Si ratio of 1.70 is assumed, then the following water content is obtained, (CaO)_1.70_SiO_2_(H_2_O)_3.65_ resulting in a density value of 2.06 g cm^−3^. The C-S-H water content, including gel water, ∼3.7–4.0, and the density value ∼2.1 g cm^−3^ justify the choice of the CSH-II model for the thermodynamic modelling study, sections 2.4 and 3.6.

The variation of the electron density (and so volumetric mass density) of Op and Ip C-S-Hs is worth analysing in detail. The electron-density variation of Ip volumes in the PC–CC blend paste is larger than that of Op regions. There are regions of Ip with electron densities lower than 0.57 e Å^−3^ and so mass densities lower than 1.7 g cm^−3^, see Figs. 8 ▸(*a*) and 8 ▸(*b*). The study of more than ten independent volumes for each type of gel gave an average electron-density value of 0.61 and 0.67 e Å^−3^ for Ip and Op, respectively. Furthermore, we noticed that most Ip volumes have a bimodal distribution of electron density. Taken all together, and at least for this sample, the association between high density C-S-H gel and Ip C-S-H, previously suggested by S˘milauer & Bittnar (2006 ▸) and Chen *et al.* (2010 ▸), must be ruled out.

The situation concerning the water content and density of C-S-H gel in PC–CC blend paste is more complicated because of the observed electron-density variability. The average electron density is 0.64 e Å^−3^, see Table 4 ▸, slightly smaller than that of the gel in the neat PC paste, 0.66 e Å^−3^. Therefore, the average density of the C-S-H gel for this blend must be slightly lower than that of neat PC paste, but it is not possible to measure with accuracy as the presence of carbonates *etc.* does not allow one to measure the water content from the average absorption value. Under the approximation of total water molecules of 4.0, the estimated density is ∼2.05 g cm^−3^. Therefore, it is still considered high density C-S-H (Jennings, 2008 ▸). On the other hand, the average electron density of the C-S-H gel for PC–FA blend paste is significantly lower, 0.56 e Å^−3^, see Table 5 ▸. Under the approximation of an overall water molecule content of 6.0, the estimated density is ∼1.77 g cm^−3^ which is considered a low density C-S-H region according to the CM-II colloidal model (Jennings, 2008 ▸). This lower value of the density of C-S-H is totally in line with the higher w/c ratio estimated in Section 3.1. It is noted that the expected Ca/Si ratio in the C-S-H gel for PC–FA should be smaller than 1.8 (Lothenbach *et al.*, 2011 ▸; Deschner *et al.*, 2012 ▸) but the overlapping of the electron densities of C-S-H and ettringite, see Fig. 3 ▸(*c*), does not allow one to extract accurate information.

A related study was undertaken for the Fe–Al siliceous hydrogarnet gel, Ca_3_(Fe,Al)_2_(SiO_4_)_y_(OH)_12_−_4y_·nH_2_O. This is the main iron-containing phase in mature PC pastes (Dilnesa, Wieland *et al.*, 2014 ▸; Vespa *et al.*, 2015 ▸). For this component, the situation is more complex than that of C-S-H gel, as there are three degrees of freedom (instead of two): (i) Al/Fe ratio, (ii) (SiO_4_)^4−^/(OH)^−^ ratio and (iii) overall water content because of the possible presence of gel pore water between the hydrogarnet nanoparticles. So, assuming the stoichiometry already reported, for Ca_3_FeAl(SiO_4_)_0.84_(OH)_8.64_ the resulting density value is ρ = 2.52 g cm^−3^. On the other hand for a simplified stoichiometry, Ca_3_FeAl(SiO_4_)(OH)_8_, the average electron density led to a density of 2.53 g cm^−3^, which shows that the obtained density is only marginally affected by the assumed stoichiometry. The density measured by PXCT is much lower than that determined for a well crystallized phase, 3.09 g cm^−3^ (Dilnesa, Lothenbach *et al.*, 2014 ▸), underlying the poorly ordered nature of Fe–Al siliceous hydrogarnet formed in PC pastes.

To end this section, it is worth noting that amorphous silica from FA (component phase 6) has mainly round particles as shown in Fig. 5 ▸(*a*). The density of the unreacted silica-rich particles is ∼2.56 g cm^−3^, calculated from the average electron-density value, 0.77 e Å^−3^, with an assumed chemical composition of SiO_2_. It is noted that this assumption is an approximation because of the large Al_2_O_3_ content of the FA, 26.4 wt%, see Table S3. The density of FA depends upon its composition. Reported average density values of F-class and C-class are 2.38 and 2.65 g cm^−3^, respectively (Kosmatka *et al.*, 1996 ▸). It is natural to deduce that lower density silica particles are more reactive for the pozzolanic reaction, and the unreacted fraction is that mainly composed by high density particles. For the sake of completeness, we also give here the density values of the room pressure crystalline SiO_2_ polymorphs quartz, cristobalite and tridymite, which are 2.65, 2.32 and 2.31 g cm^−3^, respectively (Chatterton & Cross, 1972 ▸).

### 3.6. PXCT tomogram segmentation: quantitative component phase analysis   

The results of the tomographic segmentation discussed just below, and renormalized after excluding air porosity are reported in Table 6 ▸. These values are compared with the initial contents which are also given in Table 6 ▸. The same results but expressed as wt% are given in Table S5. It is underlined that one of the biggest advantages of this technique is the information in the morphology of the hydrates and its spatial distribution.

**Figure 10:**
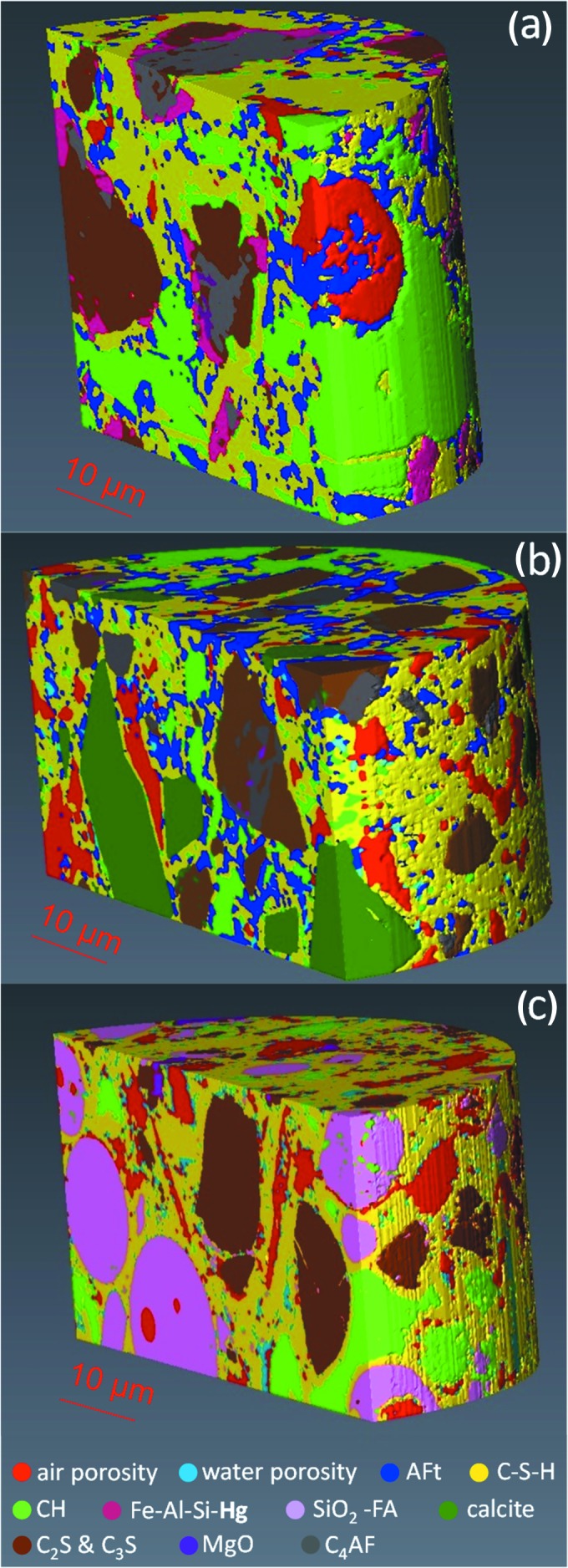
Selected views of the 3D renderings of the segmented volumes showing the components for (*a*) neat PC paste, (*b*) PC–CC blend paste and (*c*) PC–FA blend paste. Colour codes for the different component phases are given at the bottom.

Threshold-based image segmentations were performed for three large VOIs of about 2.5 × 10^4^ µm^3^ of the electron-density tomograms of the three pastes and the final results are displayed in Fig. 10 ▸. Additional views of the segmented volumes are given in Fig. S8. For neat PC paste, see Fig. 10 ▸(*a*), air porosity (defined as *n*_e_ ≤ 0.20 e Å^−3^) was 2.2 vol% and capillary pore solution (defined as 0.20 < *n*_e_ ≤ 0.40 e Å^−3^) was not found. AFt was defined between 0.40 < *n*_e_ ≤ 0.60 e Å^−3^. For the HD_C-S-H (component phase 3) and CH (component phase 4) segmentation procedure, both electron densities and attenuations were used. HD_C-S-H was defined as the component with 0.60 < *n*_e_ ≤ 0.68 e Å^−3^ and 201 < μ ≤ 339 cm^−1^ and CH was defined by 0.64 < *n*_e_ ≤ 0.72 e Å^−3^ and 339 < μ ≤ 524 cm^−1^. Fe–Al–Si–**Hg** (component phase 5) was defined as 0.72 < *n*_e_ ≤ 0.85 e Å^−3^ and it is mainly associated to component phase 11, C_4_AF, see Fig. 10 ▸(*a*). C_3_S and C_2_S were segmented together, because of their similar electron densities (and absorption values), using the 0.85 < *n*_e_ ≤ 1.02 e Å^−3^ range. For MgO and C_4_AF segmentation, electron densities and attenuations were also used. MgO was defined by 1.02 < *n*_e_ e Å^−3^ and μ ≤ 333 cm^−1^ and C_4_AF was defined by 1.02 < *n*_e_ e Å^−3^ wih μ > 333 cm^−1^. The spatial distribution of Fe–Al–Si–**Hg** (pink) is mainly surrounding C_4_AF (grey), as expected. AFt (blue) crystallizes with small particles sizes ranging from 0.5 to 2 µm. Conversely, portlandite (light green) ranges from very small to very large particles, from smaller than 0.5 µm to larger than 15 µm, respectively.

For PC–CC blend paste, see Fig. 10 ▸(*b*), air porosity was 9.5 vol% and the vol% of the other phases are reported in Table 6 ▸, renormalized after excluding air porosity. Here, a set of component phases were segment defined as 0.40 < *n*_e_ ≤ 0.57 e Å^−3^ which include AFt and AFm type phases. For the segmentation of the HD_C-S-H/CH pair, HD_C-S-H was defined by 0.57 < *n*_e_ ≤ 0.70 e Å^−3^ and μ ≤ 346 cm^−1^ while CH was defined by 0.65 < *n*_e_ ≤ 0.75 e Å^−3^ and μ > 346 cm^−1^. Calcite was defined as 0.75 < *n*_e_ ≤ 0.85 e Å^−3^. C_3_S and C_2_S were segmented together and MgO and C_4_AF were segmented independently as indicated above. Not only does this blend paste contain less portlandite than the neat PC paste, 10.2 vol% versus 17.8 vol%, but the size of the portlandite particles (light green) are significantly smaller. It is also worth noting that the straight edges of calcite crystals (dark green) are mainly surrounded by C-S-H gel (yellow).

For PC–FA blend paste, see Fig. 10 ▸(*c*), air porosity was 14.1 vol% and the vol% of the other components are reported in Table 6 ▸, renormalized after excluding air porosity. A set of component phases were segmented between 0.40 < *n*_e_ ≤ 0.63 e Å^−3^ including AFt and LD_C-S-H gel (component phases 1 and 2). Then, CH was defined as 0.63 < *n*_e_ ≤ 0.72 e Å^−3^. We recall that the low electron density of C-S-H gel allows the segmentation of these two components, C-S-H and CH, without the need for using the attenuations as there is no strong overlapping, see Fig. 3 ▸(*c*). FA was defined as 0.72 < *n*_e_ ≤ 0.88 e Å^−3^ and C_2_S as 0.88 < *n*_e_ ≤ 1.02 e Å^−3^. Finally, MgO was defined as previously reported. The morphology of unreacted FA (light pink) is spherical, as expected. It is noted that the amount of cenosphere (hollow FA particles) content is very small as displayed in Fig. 10 ▸(*c*), shown by the air spheres (red) within the SiO_2_–Al_2_O_3_ FA spherical particles. Also evident in this figure is the presence of air porosity with thin-plaque morphology (red) that is likely to have been formed from the dissolution of portlandite in the hardened state as discussed above.

The determined and expected component content for the hydrated phases will be discussed in the next section. Here, it is highlighted that the added calcite content in the PC–CC blend was 12.4 vol% (plus 0.9 vol% from PC sample) and the determined value by segmentation was 15.8 vol%, see Table 6 ▸. We justify this apparent disagreement because the segmented value for CaCO_3_ (0.75 < *n*_e_ ≤ 0.85 e Å^−3^) also included any Fe–Al–Si–**Hg**. We recall that the electron-density value of ∼0.77 e Å^−3^ is within the range of the former and that these phases cannot be disentangled by using the attenuation coefficients as they are too close. The μ values for Fe–Al–Si–**Hg** and CaCO_3_ are ∼350 and 415 cm^−1^, respectively.

The initial FA content in the PC–FA blend was 17.9 vol% and the determined value by segmentation was 16.9 vol%, see Table 6 ▸. A simplistic comparison will lead to the estimation of about 5% reaction degree of FA caused by the pozzolanic reaction. However, this assumption does not hold as the segmented value for FA (0.72 < *n*_e_ ≤ 0.88 e Å^−3^) also contains some Fe–Al–Si–**Hg**, *n*_e_ ≃ 0.77 e Å^−3^. In fact, thermodynamical modelling (see next section) indicates ∼8.6 vol% of Fe–Al–Si–**Hg** should be formed in these conditions. So assuming that the segmented FA component contains some Fe–Al–Si–**Hg** content, then a reaction degree of ∼40% is obtained. We acknowledge that the assumptions employed here are subject to large errors and any reaction degree between 25% and 55% is compatible with our findings. However, both the overall segmented content of FA and the decrease in the segmented portlandite content, see below, point towards a pozzolanic reaction degree close to 30%. This reaction degree is in good agreement with the observations of De Weerdt *et al.* (2011 ▸).

Finally, it is worth discussing briefly the spatial resolution in the segmented tomograms. Spatial resolution is in general difficult to estimate. In this work, the resolution in the raw data, determined by Fourier shell correlation, was better than 80 nm in the electron-density tomograms and around 250 nm in the absorption tomograms. How this is propagated through the data analysis into the segmented volumes, convoluted with the partial volume effect, is difficult to assess. To contribute to this analysis, Fig. 9 ▸(*a*) was selected where the porosity volume surrounding the FA particle was shown to have about 200 nm width. This could be segmented, as observed in the light blue volumes surrounding the spherical particle (pink) in Fig. 9 ▸(*c*). Hence, a spatial resolution close to 200 nm in the segmented tomograms is estimated. However, we acknowledge that this procedure is just an approximation and further work is needed to establish figure(s) of merit for determining the real spatial resolution in segmented tomograms

### 3.7. Thermodynamic modelling: quantitative component phase prediction   

The hydration reactions of the neat PC cement were simulated for up to 5 months in order to compare the results with those obtained by PXCT. As the w/c mass ratio was previously estimated to be close to 0.27, this value was used for the thermodynamic modelling. The quantitative phase assemblage obtained by the Rietveld methodology for the anhydrous PC sample was used as input data and the Parrot and Killoh model was used to simulate the amount of reacted cement (Lothenbach *et al.*, 2008 ▸) with the hydration time. The hydration evolution was followed for up to 5 months, see Fig. S9. It is noted that this comparison should be exercised with care as some approximations and systematic errors can have a role to play. Firstly, the w/s ratio estimated by PXCT is subject to error and it is not necessarily constant along such thin capillaries. Secondly, the ultrasound treatment can alter the anhydrous phase assemblage. Thirdly, the segmentation procedure groups all the component phases with a similar electron density (and attenuation value). Finally, the thermodynamic modelling also contains approximations. In any case, the comparison is given for a semi-quantitative validation. Hence, Table 7 ▸ shows the phase assemblage, in volume percentage, for the neat PC cement (including the added water) and that for the hydrated PC cement after 5 months of hydration obtained by thermodynamic modelling and by PXCT (excluding air porosity). The agreement between both methodologies is reasonable considering the complexity of the sample. The thermodynamic modelling analysis shows that the reaction degree is slightly larger than that observed by PXCT, as there are a fewer number of anhydrous components after 5 months. The pore solution content predicted in the thermodynamic modelling is very low which is in agreement with the absence of porosity found in the PXCT study. Moreover, the precipitation of C-S-H and CH matched, within the margin of error, the experimental results obtained by PXCT. In addition, it is worth mentioning the relatively good agreement for Fe–Al siliceous content. Finally, the largest disagreement is related to the content of ettringite. This could be because of a number or reasons including that thermodynamic modelling assumes all sulfates crystallize as ettringite but this anion can be partly incorporated into other phases.

The same type of study was performed for the PC–CC blend paste. The w/s mass ratio of the PXCT studied region was 0.27. Table 8 ▸ shows the phase assemblage, in volume percentage, for the anhydrous PC–CC blend and for the hydrated sample after 5 months of hydration obtained by thermodynamic modelling and by PXCT (excluding air porosity). Fig. S10 displays the evolution of the phase assemblage for this paste obtained by thermodynamic modelling. The comparison of the data shown in Table 8 ▸ indicates that the fraction of unreacted phases measured by PXCT was larger than that calculated by modelling. Furthermore, greater pore solution content was calculated by modelling than found experimentally. This can be explained by a (slightly) larger w/s ratio than that estimated in Section 3.2 and also by a larger degree of reaction deduced from the Parrot and Killoh model. It is worth mentioning that the measured calcite content by PXCT was larger than the initial value, 15.8 vol% versus 13.3 vol%, because it also contains the amount of formed Fe–Al–Si–**Hg**, see Table 8 ▸. The agreement between the modelled and determined C-S-H contents is very satisfactory. However, the portlandite content determined by PXCT was lower than that modelled and also than that expected from a calcite dilution of the neat PC study shown above. This result requires further investigation or confirmation.

For the PC–FA blend paste, the w/s ratio of the studied region was 0.30. The phase assemblage’s evolution for the paste obtained by thermodynamic modelling is shown in Fig. S11, and Table 9 ▸ reports the phase assemblages modelled after 5 months of hydration and measured by PXCT (excluding air porosity). The measured unreacted FA content by PXCT was 16.9 vol% larger than the initial value because it also contains the amount of Fe–Al–Si–**Hg** formed. Chiefly, portlandite content measured by PXCT, 11.1 vol%, is lower than the value determined for the neat PC shown above, ∼18 vol%, and after dilution with the added FA, ∼14 vol%. This is indirect proof of a pozzolanic reaction which is even clearer when considering that the unreacted fraction of Portland cement is lower in the PC–FA blend. However, the PXCT determined value, 11.1 vol%, is larger than the modelled value, 5.8 vol%, which is justified by the set of approximations used in both approaches.

## 4. Conclusions   

Despite the relatively low spatial resolution, ∼250 nm, and s/n ratio in the absorption tomograms compared with the electron-density tomograms, the combined use of δ and β datasets is key to identifying components with quite similar electron densities but different attenuation coefficients, and chiefly in the segmentation procedure for obtaining accurate analyses for these complex mixtures. The main examples in this study were a C-S-H gel and crystalline Ca(OH)_2_ pair and an MgO and Ca_4_Al_2_Fe_2_O_10_ pair. Having a high spatial resolution in the electron-density tomograms, better than 80 nm, and quantitative contrast for the different mineral components allow one to minimize partial volume effects and measure properties at length scales of a few hundreds of nanometres. Examples of this include: (i) accurate segmentation of hydrated component phases in neat PC paste; (ii) identification and quantification Fe-hydrogarnet gel precipitated on the surfaces of Ca_4_Al_2_Fe_2_O_10_; and (iii) distinguishing between inner-product and outer-product C-S-H gels. The high spatial resolution also allows one to segment crystalline portlandite which appears in quite different microstructures ranging from bulk irregular regions with sizes larger than 20 µm to highly anisotropic plaques with lengths from 5 to 10 µm and thicknesses of ∼0.5 µm, interspersed with C-S-H gel as previously observed (see for instance, Trtik *et al.*, 2012 ▸).

The density and water content of unaltered high-density C-S-H gel of a neat Portland cement paste have been measured to be ρ = 2.11 g cm^−3^ and *n* = 3.96 for an assumed Ca/Si molar ratio of 1.80, *i.e.* (CaO)_1.8_SiO_2_(H_2_O)_4.0_. These density and water content values are in line with results previously reported using other techniques (Allen *et al.*, 2007 ▸; Jennings, 2008 ▸; Thomas *et al.*, 2010 ▸; Muller *et al.*, 2013 ▸) but obtained here without any sample treatment. For the PC–calcite blend paste, the smaller alite reaction degree has allowed one to distinguish inner-product C-S-H and outer-product C-S-H gels. The electron-density variations within the inner-product C-S-H, which can be as high as 25% (from 0.53 to 0.67 e Å^−3^), are larger than those of outer-product C-S-H, from 0.59 to 0.73 e Å^−3^. Furthermore, it is demonstrated that inner-product C-S-H cannot be directly associated with high-density C-S-H. For the first time, and to the best of our knowledge, the density of Fe-hydrogarnet gel has been measured [assumed stoichiometry Ca_3_FeAl(SiO_4_)_0.84_(OH)_8.64_] as ρ = 2.52 g cm^−3^, which is the main hydrated iron-containing phase in Portland cement pastes. It is highlighted that a 6.4 vol% of amorphous Fe–Al siliceous hydrogarnet has been directly measured in the presence of 41.1 vol% of poorly crystalline C-S-H gel.

Finally, for the PC–FA blend paste, the FA reaction degree measured after five months was not large, ∼30%. Some regions of the tomogram have air porosity with straight edges and we hypothesized that these regions are formed by portlandite dissolution at the late stages of hydration, when the sample is hardened, caused by a pozzolanic reaction with the fly ash. In the absence of liquid phase to fill these pores, this process leaves a negative print of the initial portlandite particles when it takes place in a hardened paste. For this paste, cracks with the dimensions of a few hundreds of nanometres have been observed, likely to be caused by shrinkage. This shrinkage, which is not detected in the other two pastes, is hypothesized to be the result of its larger w/c ratio.

## 5. Related literature   

The following references cited in the Supporting information: Cullity (1956 ▸); Dinapoli *et al.* (2011 ▸); Gorelick *et al.* (2011 ▸); Guizar-Sicairos *et al.* (2015 ▸); Holler, M. & Raabe, J. (2015 ▸); Holler *et al.* (2012 ▸); Howells *et al.* (2009 ▸); Huang *et al.* (2014 ▸); Kaestner *et al.* (2011 ▸); Liu & Daum (2008 ▸) and Sánchez-Herrero *et al.* (2016 ▸).

## Supplementary Material

Supporting information including tables and figures. DOI: 10.1107/S2052252519003774/ro5017sup1.pdf

Ptychographic X-ray computed tomography data for three Portland cement pastes: https://doi.org/10.5281/zenodo.2533863

## Figures and Tables

**Table 1:** Chemical stoichiometries of the component phases with the abbreviation and numbering system used in the text and in the figures, respectively

Numerical labels used in the figures	Chemical formula	Text abbreviation
1	Ca_6_Al_2_(SO_4_)_3_(OH)_12_·26H_2_O	AFt
2	∼(CaO)_1.8_ (SiO_2_)(H_2_O)_6_	LD_C-S-H
3	∼(CaO)_1.8_(SiO_2_)(H_2_O)_4_	HD_C-S-H
4	Ca(OH)_2_	CH or Portlandite
5	∼Ca_3_FeAl(SiO_4_)_0.84_(OH)_8.64_	Fe–Al–Si–**Hg**
6	∼SiO_2_	FA
7	CaCO_3_	CC or Calcite
8	Ca_3_SiO_5_	C_3_S
9	Ca_2_SiO_4_	C_2_S
10	MgO	MgO
11	Ca_2_AlFeO_5_	C_4_AF

**Table 2:** Water to cement (and to solid) ratios estimated from the measured X-ray absorption values of the pastes; X-ray absorption values for anhydrous and paste samples are also given

Sample	Calculated average μ for anhydrous sample (cm^−1^)	Experimental average μ for hydrated sample (cm^−1^)	w/s (mass)	w/c (mass)	w/s (vol.)	w/c (vol.)	Water fraction (vol%)
PC	624.9	348.5	0.27	0.27	0.85	0.85	46.0
PC–CC	581.5	341.2	0.27	0.33	0.79	0.99	44.0
PC–FA	479.3	267.3	0.30	0.43	0.87	1.31	46.4

**Table 3:** Electron, mass densities and mass attenuation coefficients (μ) obtained by PXCT for the neat PC paste at five months of hydration; expected mass densities taken from the CIF files (Aranda, 2016 ▸) and expected μ (Henke *et al.*, 1993 ▸) are also given

Phase	Electron density (e Å^−3^) average of ten values	Electron density (e Å^−3^) full volume†	Expected electron density (e Å^−3^)	Calculated density (g cm^−3^)	Expected density (g cm^−3^)‡	Calculated μ (cm^−1^) (corrected)†	Expected μ (cm^−1^)
1, AFt	0.568 (4)	0.55	0.56	1.80 (1)	1.78	187	181.0
3, HD_C-S-H	0.657 (7)	0.64	—	2.11 (2)	—	279	—
4, CH	0.690 (6)	0.67	0.69	2.23 (2)	2.23	440	446.1
5, Fe-Al-Si-**Hg**	0.766 (8)	0.76	—	2.52 (3)	3.09	350	—
8, C_3_S	0.957 (11)	0.92	0.95	3.18 (4)	3.15	614	657.8
9, C_2_S	0.999 (4)	0.98	0.99	3.32 (1)	3.30	646	637.3
10, MgO	1.080 (10)	1.05	1.07	3.58 (3)	3.58	228	217.4
11, C_4_AF	1.080 (10)	1.05	1.10	3.66 (3)	3.73	591	566.4
Capillary	0.675 (4)	—	0.66	2.24 (1)	2.20	—	—

† Values obtained from the segmented components by *Avizo* software.

‡ The expected density values are determined from crystallographic data and so they are not available for nanocrystalline/amorphous components.

**Table 4:** Electron, mass densities and μ obtained by PXCT for the PC–CC blend paste after five months of hydration; expected mass densities taken from the CIF files (Aranda, 2016 ▸) and expected μ (Henke *et al.*, 1993 ▸) are also given

Phase	Electron density (e Å^−3^) average of ten values	Electron density (e Å^−3^) full volume†	Expected electron density (e Å^−3^)	Calculated density (g cm^−3^)	Expected density (g cm^−3^)‡	Calculated μ (cm^−1^) (corrected)†	Expected μ (cm^−1^)
1, Monocarbo, AFt pore solution	0.45 (3)	0.48	—	1.36 (9)	—	193	—
3, HD_C-S-H	0.64 (1)	0.63	—	∼2.05	—	228	—
4, CH	0.698 (7)	0.68	0.69	2.23 (2)	2.23	464	446.1
7, CC	0.826 (4)	0.80	0.82	2.75 (1)	2.71	411	415.2
8, C_3_S	0.963 (4)	0.93	0.95	3.20 (1)	3.15	649	657.8
9, C_2_S	0.998 (8)	0.99	0.99	3.32 (3)	3.30	639	637.3
10, MgO	1.062 (16)	1.06	1.07	3.52 (5)	3.58	211	217.4
11, C_4_AF	1.062 (16)	1.06	1.1	3.60 (5)	3.73	566	566.4
Capillary	0.674 (6)	—	0.66	2.24 (2)	2.20	—	—

† Values obtained from the segmented components by *Avizo* software.

‡ The expected density values are determined from crystallographic data and so they are not no available for nanocrystalline/amorphous components.

**Table 5:** Electron, mass densities and μ obtained by PXCT for the PC-FA blend paste after five months of hydration; expected mass densities taken from the CIF files (Aranda, 2016 ▸) and expected μ (Henke *et al.*, 1993 ▸) are also given

Phase	Electron density (e Å^−3^) average of ten values	Electron density (e Å^−3^) full volume†	Expected electron density (e Å^−3^)	Calculated density (g cm^−3^)	Expected density (g cm^−3^)‡	Calculated μ (cm^−1^) (corrected)†	Expected μ (cm^−1^)	
Pore solution	0.32 (5)	—	0.33	1.0 (2)	1.0	—	—	
1,2 LD_C-S-H & AFt	0.56 (2)	0.55	—	∼1.77	—	**228**	—	
4, CH	0.689 (9)	0.67	0.69	2.23 (3)	2.23	434	446.1	
6, FA	0.77 (2)	0.75	—	2.56 (5)	—	**218**	—	
9, C_2_S	0.999 (5)	0.97	0.99	3.32 (2)	3.30	634	637.3	
10, MgO	—	1.06	1.07	3.55	3.58	221	217.4	
Capillary	0.672 (5)	—	0.66	2.23 (2)	2.20	—	—	

† Values obtained from the segmented components by *Avizo* software.

‡ The expected density values are determined from crystallographic data and so they are not available for nanocrystalline/amorphous components.

**Table 6:** Volume percentages for the cement pastes at five months of hydration renormalized after excluding air porosity determined by tomographic segmentation The corresponding values for *t* = 0 are given to highlight the evolution of the hydrates. The *t* = 0 values were obtained from the Rietveld quantitative phase analysis for the anhydrous cements and they were renormalized taking into account the added water.

Phase	PC (*t*_0_)	PC	PC–CC (*t*_0_)	PC–CC	PC–FA (*t*_0_)	PC–FA
Capillary water	46.0	—	44.0	3.0	46.4	13.4
1, C-S-H	—	41.1	—	28.2	—	48.0
2, AFt/AFm	—	11.5	—	17.8	—	
4, Portlandite	—	17.8	—	10.2	—	11.1
5, Fe–Al–Si–**Hg**	—	6.4	—	—	—	—
6, FA	—	—	—	—	17.9	16.9
7, CC	1.1	—	13.3	15.8	0.7	—
8,9 C_3_S + C_2_S	33.0 + 10.2	18.8	26.7 + 8.2	20.3	21.8 + 6.7	9.9
10, MgO	0.6	0.7	0.5	0.7	0.4	0.7
11, C_4_AF	6.6	3.8	5.3	4.0	4.3	—

**Table 7:** Phase assemblage for the neat PC paste and after five months of hydration (by thermodynamic simulation with *GEMs* and by PXCT)

Phase	Vol % (initial) with water	Vol % (*GEMs*)	Vol % (PXCT)
C_3_S	33.0	5.4	18.8
C_2_S	10.2	6.3	
C_4_AF	6.6	3.8	3.8
MgO	0.6	0.4	0.7
C_3_A†	1.5	0.4	—
Bass	1.0	—	—
CaCO_3_	1.1	0.9	—
Fe–Al–Si–**Hg**	—	4.0	6.4
Portlandite	—	20.1	17.8
C-S-H gel	—	35.3	41.1
Hydro­talcite (2.01 g cm^−3^)	—	1.3	—‡
AFt	—	18.6	11.5
Pore solution	46.0	3.6	0

† C_3_A correspond to Ca_3_Al_2_O_6_

‡ Hydro­talcite, if present, could not be independently segmented as its electron density and attenuation values are too close to those of C-S-H gel.

**Table 8:** Phase assemblage for the PC–CC blend paste and after five months of hydration (by thermodynamic simulation with *GEMs* and by PXCT)

Phase	Vol % (initial) with water	Vol % (*GEMs*)	Vol % (PXCT)
C_3_S	26.7	3.0	20.3
C_2_S	8.2	4.1	
C_4_AF	5.3	2.3	4.0
MgO	0.5	0.4	0.7
C_3_A	1.2	0.2	—
Bass	0.8	—	—
CaCO_3_	13.3	13.8	15.8
Fe–Al–Si–**Hg**	—	4.1	—†
Portlandite	—	17.0	10.2
C-S-H	—	29.2	28.2
Hydro­talcite	—	1.2	—
AFt	—	14.7	17.8
Pore solution	44.0	10.1	3.0

† Fe–Al–Si–**Hg** could not be independently segmented as its electron density and attenuation values are too close to those of calcium carbonate.

**Table 9:** Phase assemblage for the PC–FA blend paste and after five months of hydration (by thermodynamic simulation with *GEMs* assuming 30% reaction degree of the FA, and by PXCT)

Phase	Vol % (initial) with water	Vol % (*GEMs*)	Vol % (PXCT)
C_3_S	21.8	1.5	9.9
C_2_S	6.7	2.4	
C_4_AF	4.3	1.2	—
MgO	0.4	0.2	0.7
C_3_A	1.0	—	—
Bass	0.7	—	—
CaCO_3_	0.7	—	—
FA	17.9	14.2	16.9
Fe–Al–Si–**Hg**	—	8.6	—†
Portlandite	—	5.8	11.1
Monocarbonate (2.22 g cm^−3^)	—	4.1	—
C-S-H	—	35.8	48.0
Hydro­talcite	—	1.5	
AFt	—	13.1	
Pore solution	46.4	11.7	13.4

† Fe–Al–Si–**Hg** could not be independently segmented as its electron density and attenuation values are too close to those of (unreacted) FA.
