# Supplementary material for: Quantitative disentanglement of nanocrystalline phases in cement pastes by synchrotron ptychographic X-ray tomography
Source: IUCrJ. 2019 Apr 27;6(Pt 3):473–91. doi: 10.1107/S2052252519003774 (PMC6503921; doi:10.1107/S2052252519003774)
Supplement: Supplementary file 1 [file m-06-00473-sup1.pdf]

IUCrJ

Volume 6 (2019)

Supporting information for article:

Quantitative disentanglement of nanocrystalline phases in cement pastes by synchrotron ptychographic X-ray tomography

Ana Cuesta, Ángeles G. De la Torre, Isabel Santacruz, Ana Diaz, Pavel Trtik, Mirko Holler, Barbara Lothenbach and Miguel A.G. Aranda

Extended Materials and Methods

Data availability. The six tomograms, reconstructed 'raw' data in tiff format, used in this article can be freely accessed on Zenodo at https://doi.org/10.5281/zenodo.2533863, and used under the Creative Commons Attribution license. The information about the code used for the upload files is the following:

PC sample:

tomo_beta_S02536_to_S03341_Hann_freqscl_0.35_0xxx

tomo_delta_S02536_to_S03341_Hann_freqscl_1.00_0xxx

PC-CC sample:

tomo_beta_S04692_to_S06001_Hann_freqscl_0.35_0xxx

tomo_delta_S04692_to_S06001_Hann_freqscl_1.00_0xxx

PC-FA sample:

tomo_beta_S03351_to_S04661_Hann_freqscl_0.35_0xxx

tomo_delta_S03351_to_S04661_Hann_freqscl_1.00_0xxx

Table S1 XRF analysis for the neat PC

Oxides	wt%
SiO_2	20.3
Al_2O_3	5.0
Fe_2O_3	3.3
CaO	63.0
MgO	1.2
SO_3	3.5
K_2O	1.0
Na_2O	0.3
LoI*	2.3

*Loss on ignition

Table S2 Rietveld Quantitative Phase Analysis (RQPA) for the neat PC

phase	wt%
C_3S	59.2
C_2S	19.5
C_4AF	14.2
C_3A	2.5
MgO	1.2
Bassanite	1.6
$CaCO_3$	1.7

Table S3 XRF analysis for the Fly Ash. Data taken from literature (Sánchez-Herrero *et al.*, 2016).

Oxides	wt%
Al_2O_3	26.40
CaO	4.53
Fe_2O_3	7.45
K_2O	3.56
SiO_2	52.70
MgO	1.93
P_2O_5	0.28
TiO_2	0.96
MnO	0.05
Na_2O	0.53
LoI	1.60

*Loss on ignition

Table S4 Rietveld Quantitative Phase Analysis (RQPA) for the Fly Ash

phase	wt%
Mullite, $Al_{4.5}Si_{1.5}O_{9.75}$	8.6
Hematite	0.3
MgO	0.3
Magnesio-Ferrite	1.0
Quartz - SiO_2	4.6
ACn	85.3

Table S5 Phase contents (%wt) for the cements pastes at 5 months of hydration renormalized after excluding air porosity determined by tomographic segmentation.

<table>
<tr><th>Phase</th><th>PC</th><th>PC-CC</th><th>PC-FA</th></tr>
<tr><td>Capillary water</td><td>-</td><td>1.3</td><td>6.6</td></tr>
<tr><td>1, C-S-H</td><td>35.9</td><td>24.6</td><td rowspan="2">42.2</td></tr>
<tr><td>2, AFt/AFm</td><td>8.5</td><td>10.3</td></tr>
<tr><td>4, Portlandite</td><td>16.4</td><td>9.7</td><td>12.2</td></tr>
<tr><td>5, Fe-Si-Hg</td><td>6.7</td><td>-</td><td>-</td></tr>
<tr><td>6, FA</td><td>-</td><td>-</td><td>21.4</td></tr>
<tr><td>7, CC</td><td>-</td><td>18.5</td><td>-</td></tr>
<tr><td>8,9 C_3S+C_2S</td><td>25.7</td><td>28.5</td><td>16.2</td></tr>
<tr><td>10, MgO</td><td>1.0</td><td>1.1</td><td>1.3</td></tr>
<tr><td>11, C_4AF</td><td>5.9</td><td>6.1</td><td>-</td></tr>
</table>

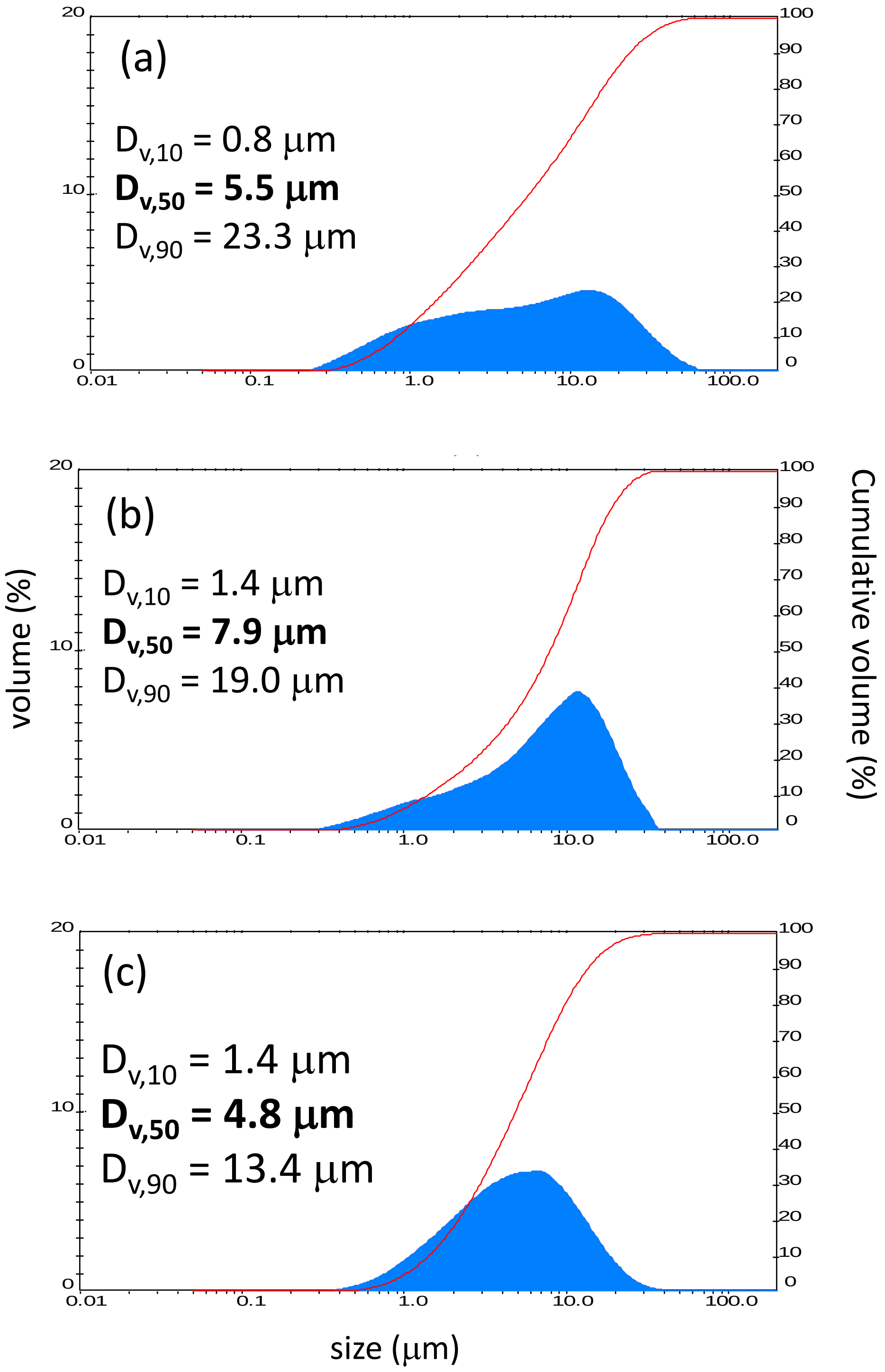

Figure S1 Particle size distribution (diameter) and cumulative measured in volume for (a) neat PC, (b) CC and (c) FA.

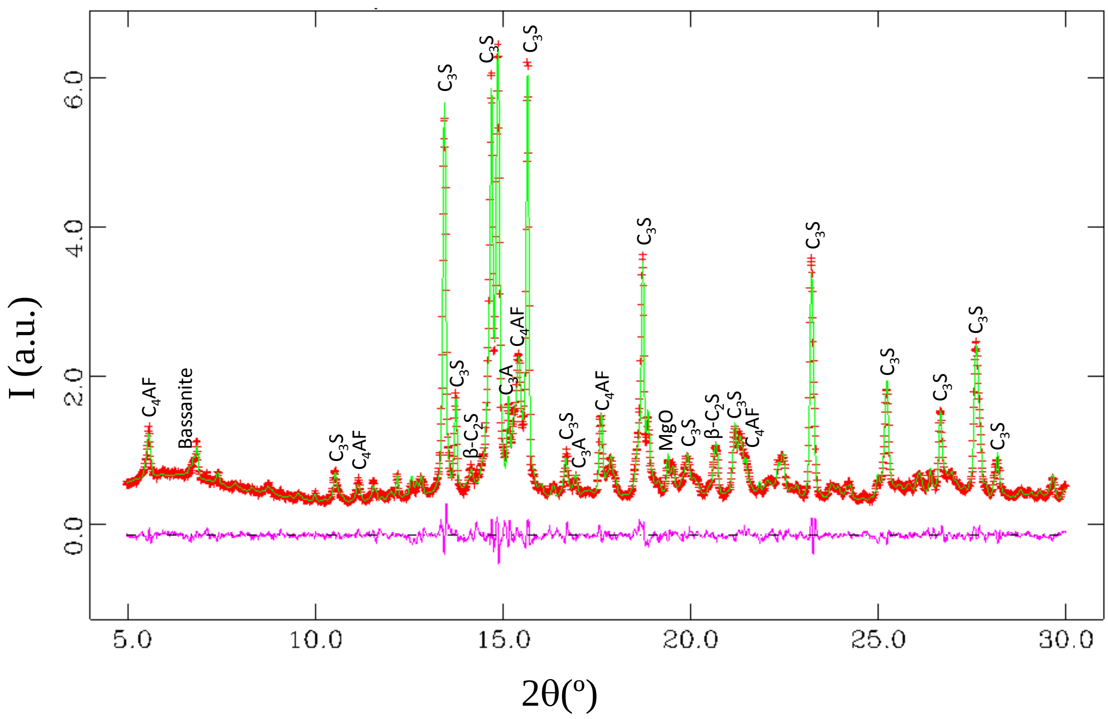

Figure S2 LXRPD Rietveld plot for plain PC. The main peaks are labeled.

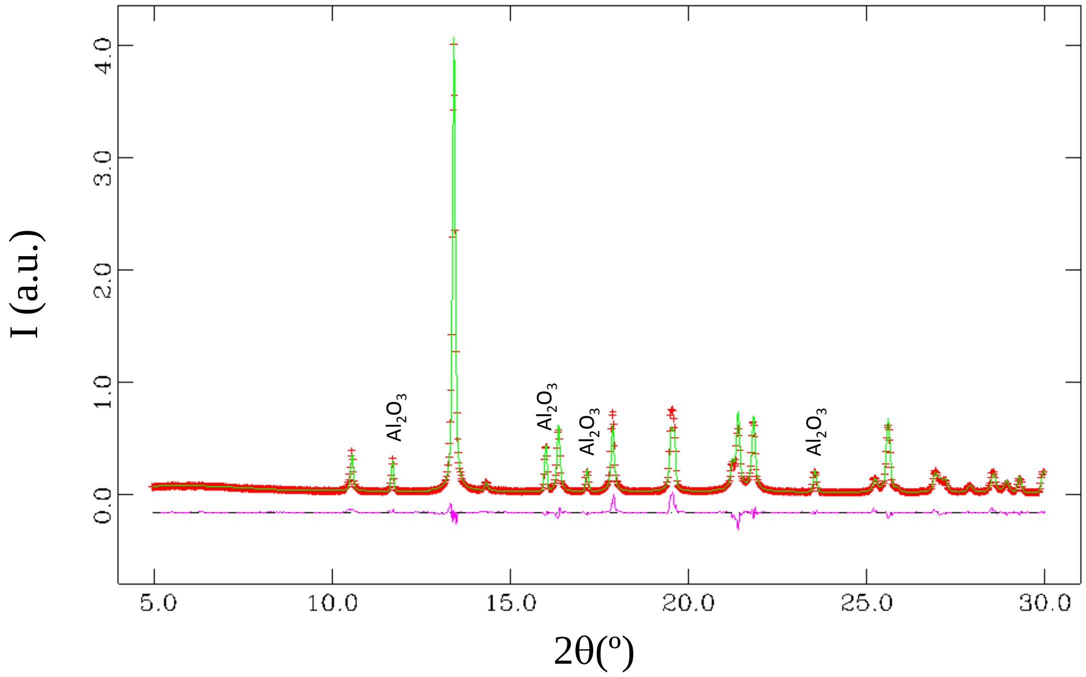

Figure S3 LXRPD Rietveld plot for $CaCO_3$. The main peaks from internal standard (α-Al_2O_3) are labeled.

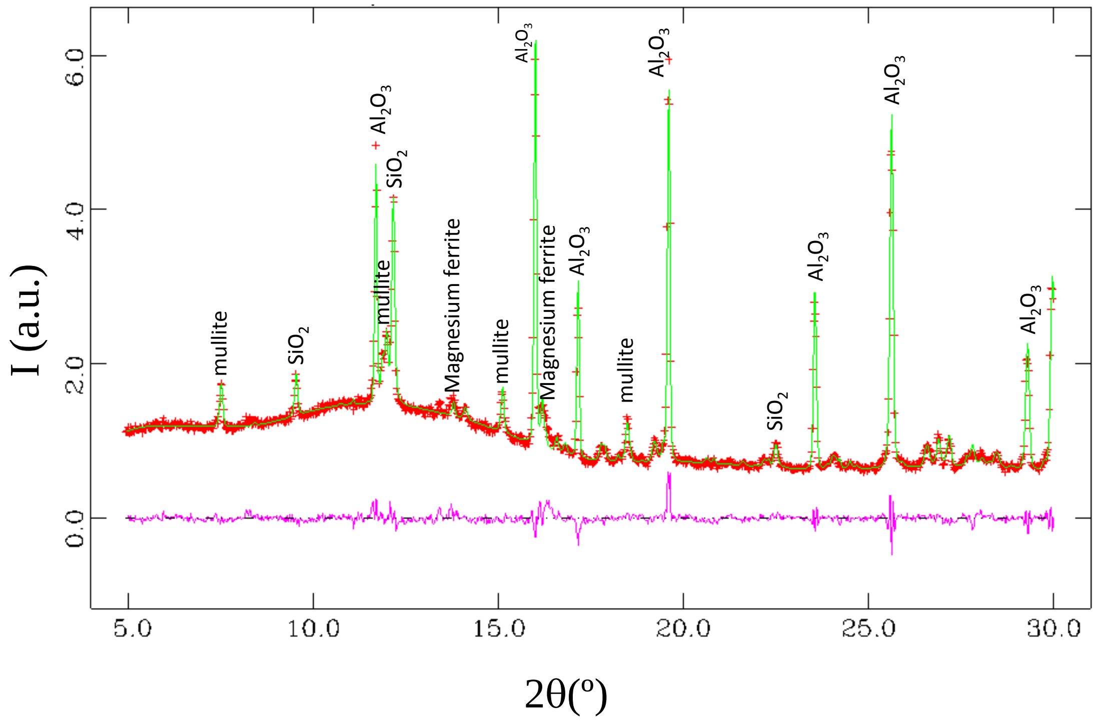

Figure S4 LXRPD Rietveld plot for Fly Ash. The main peaks are labeled.

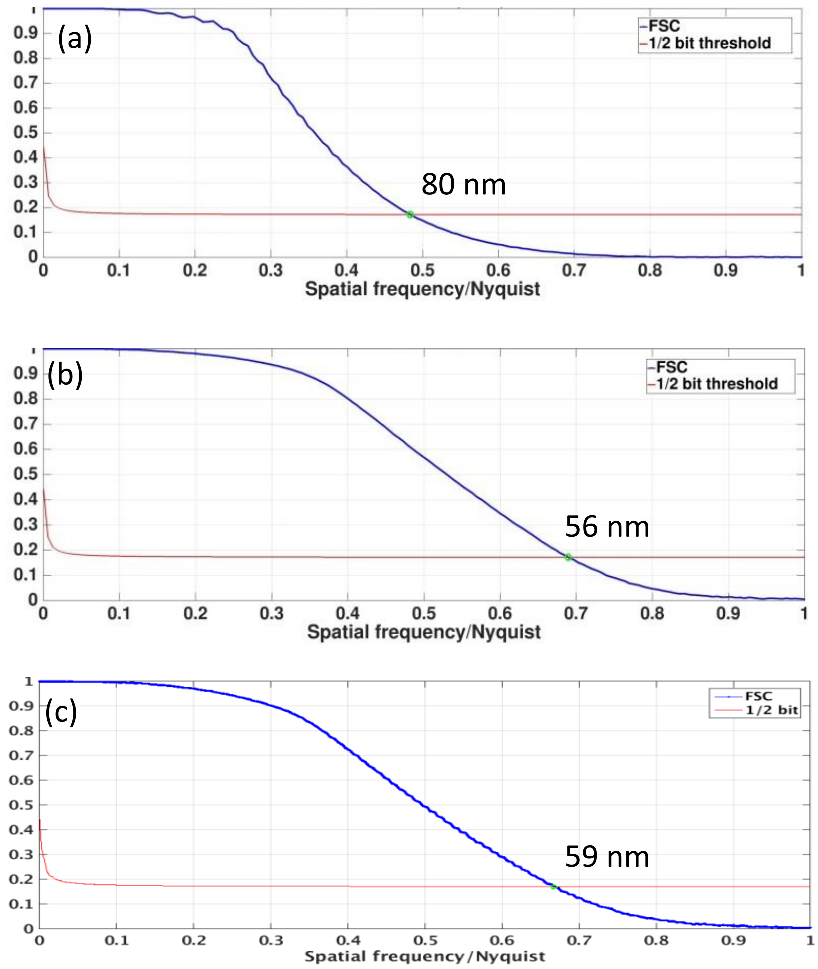

Figure S5 Fourier Shell Correlation plots from $n_e(r)$ tomograms for (a) PC paste, (b) PC with CC paste and (c) PC with FA at 5 months of hydration.

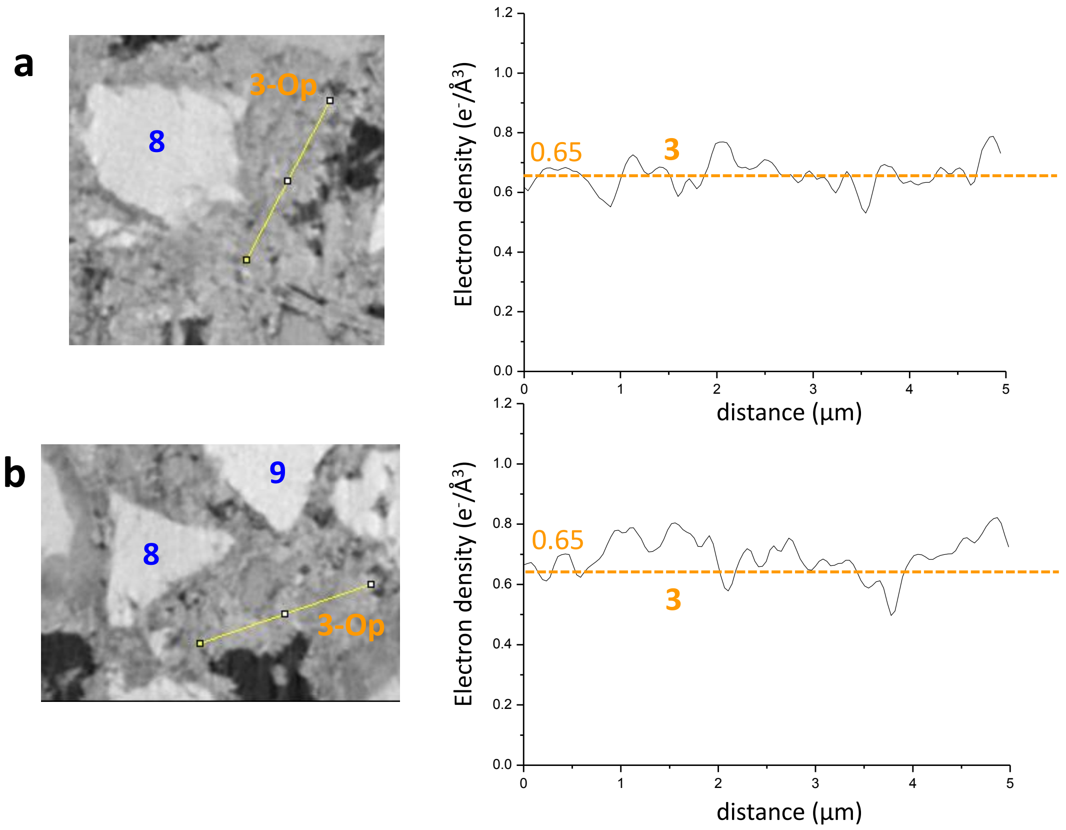

Figure S6 Two selected regions in the electron density tomogram of the PC-CC blend paste highlighting Outer product C-S-H gel. Selected lines (left) are drawn to show the variation of the electron density values along the C-S-H gels (right panels).

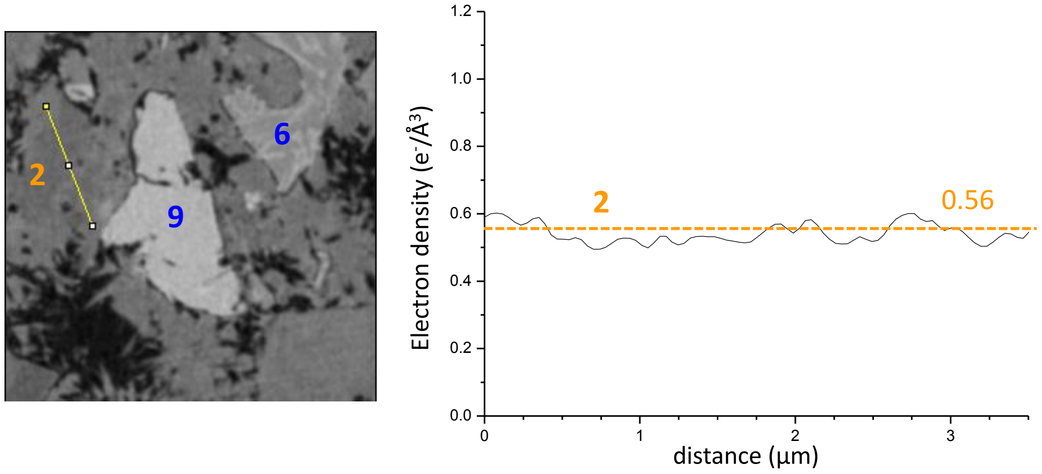

Figure S7 Selected regions in the electron density tomogram of the PC-FA blend paste highlighting low density C-S-H gel. A selected line (left) is drawn to show the variation of the electron density values along the C-S-H gel (right panel).

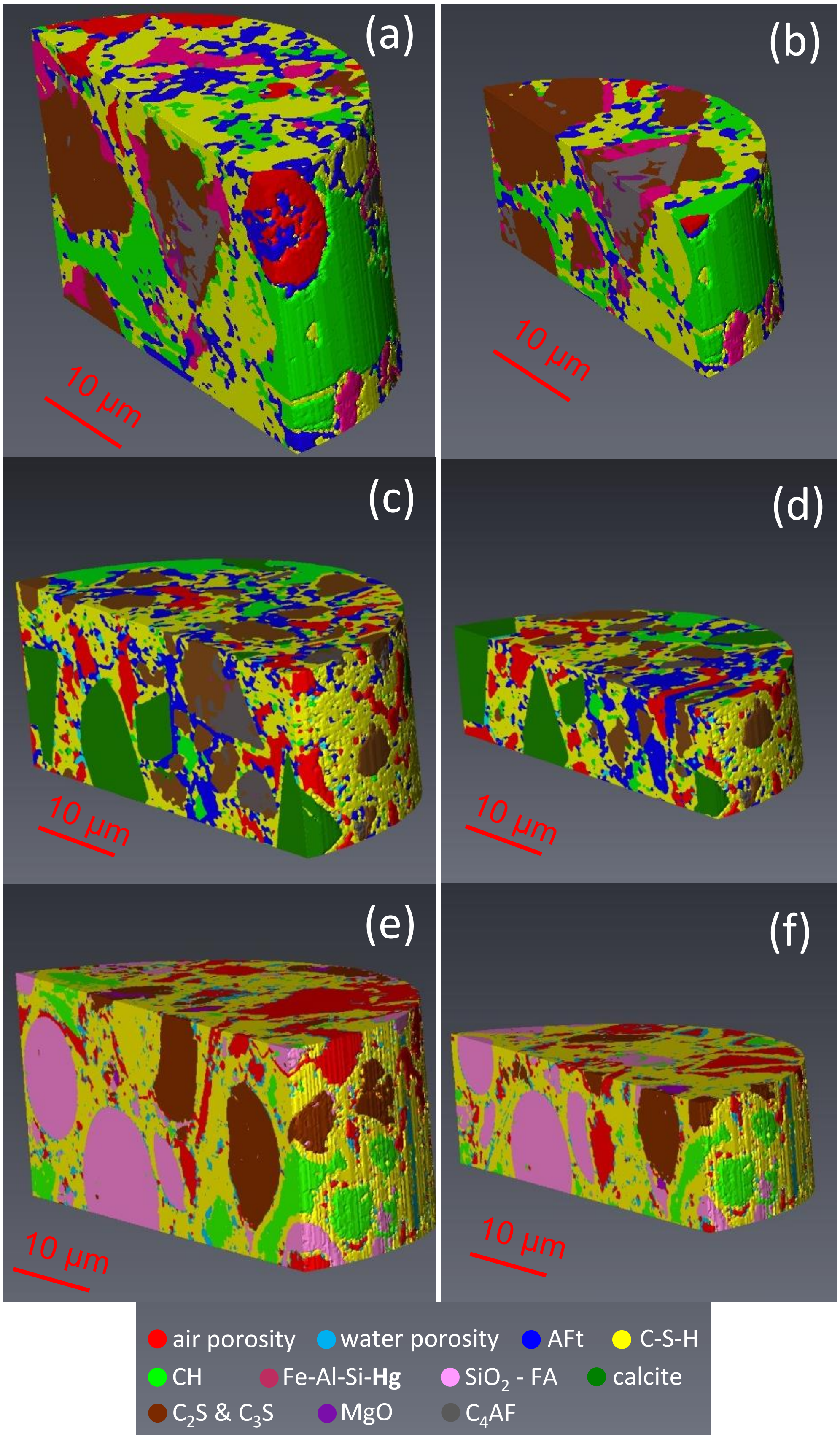

Figure S8 Additional views of the 3D renderings of the segmented volumes showing the components for (a, b) neat PC paste; (c,d) PC-CC blend paste; (e,f) PC-FA blend paste. Colour codes for the different component phases are given at the bottom.

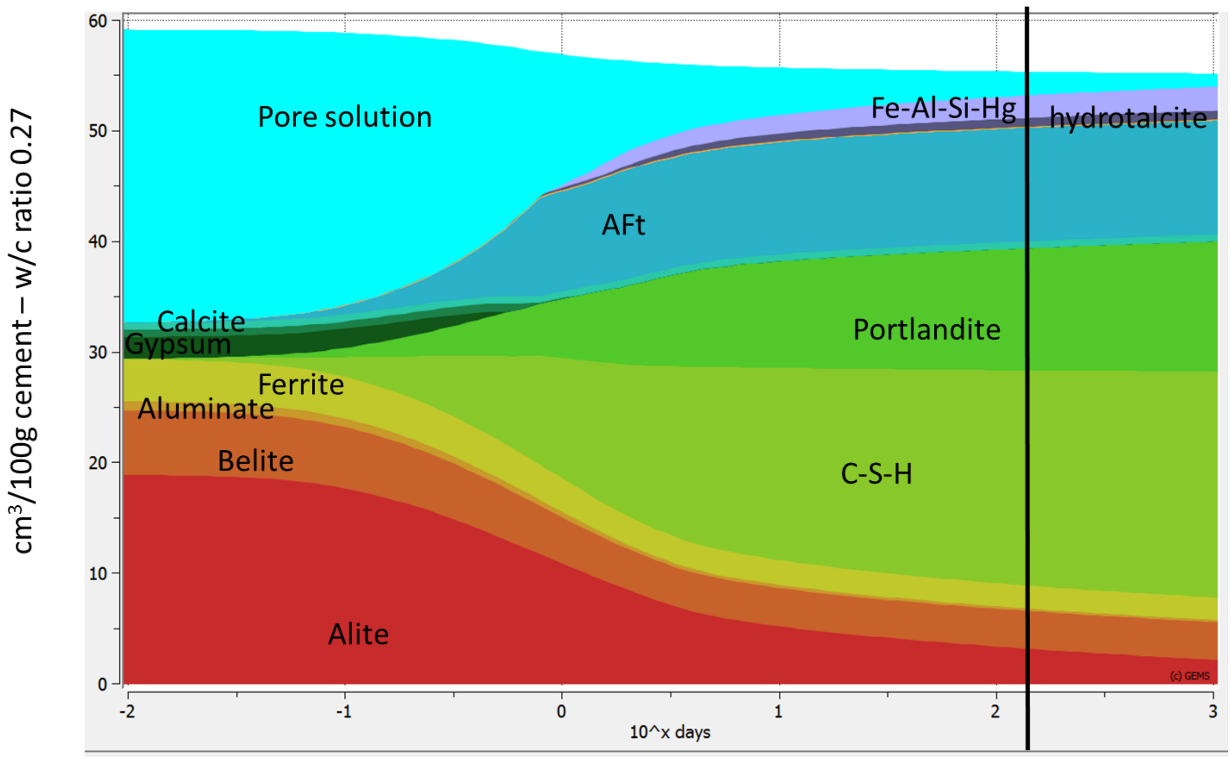

Figure S9 Volume of the different components as function of time in hydrating PC paste (w/s mass ratio = 0.27) modelled by GEMS

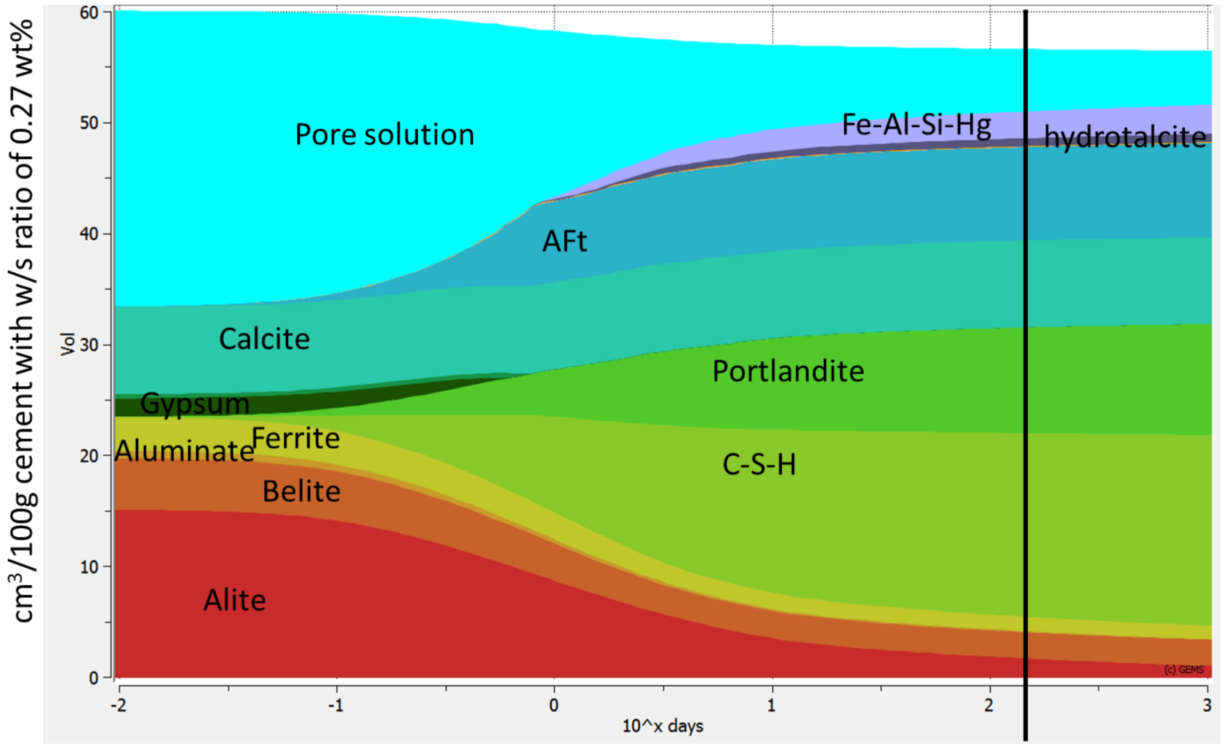

Figure S10 Volume of the different components as function of time in hydrating PC-CC paste (w/s mass ratio = 0.27) modelled by GEMS

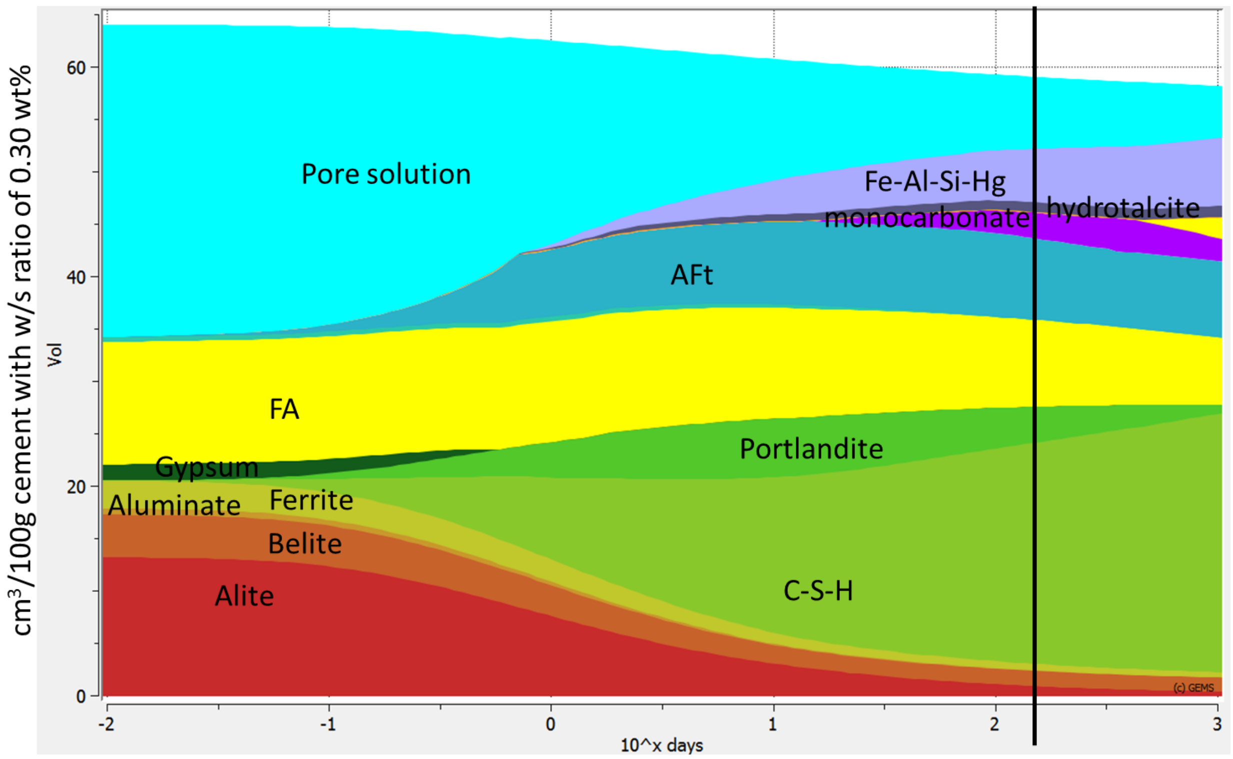

Figure S11 Volume of the different components as function of time in hydrating PC-FA paste (w/s mass ratio = 0.30) modelled by GEMS

PXCT experiments and data processing.

The PXCT experiments took place at the cSAXS beamline at the Swiss Light Source, Paul Scherrer Institute in Villigen, Switzerland, using the instrumentation previously reported in Holler *et al.* (2014, 2012), which uses laser interferometry for accurate positioning of the specimen with respect to the beam-defining optics (Holler & Raabe, 2015). A Si (111) monochromator set the X-ray photon energy to 6.2 keV. The illumination on the sample was defined by a coherently illuminated gold Fresnel zone plate (Gorelick *et al.*, 2011) of 170 μm diameter and outermost zone width of 60 nm, which for this energy had a focal length of 51 mm focal. We estimate that the flux at the sample position was about 1.3×10^{8} photons/s. The sample was placed at 2.7 mm downstream the focus, such that the illumination on the sample has a diameter of about 9 μm. For ptychographic scans, the samples were scanned at positions following a pattern based on Fermat spirals, as described in Huang *et al.* (2014). The scans covered areas of 60×30, 65×33 and 64×30 μm^2 with average step sizes of about 2.5, 3 and 3 μm, resulting in an approximate number of 290, 240 and 207 points/scan for the PC, PC-CC and PC-FA samples, respectively. Far-field diffraction patterns were recorded at each scanning position with an acquisition time of 0.08 s using an EIGER 500k detector (Dinapoli *et al.*, 2011) with a pixel size of 75 μm. The detector was placed at 7.305 m distance from the sample, such that ptychography sampling conditions were satisfied (da Silva & Menzel, 2015; Edo *et al.*, 2013). From each diffraction pattern, a region of 500×500 pixels of the detector area was used for ptychographic reconstructions, obtaining a pixel size of 38.95 nm in the reconstructed images. A flight tube flushed with He was positioned between the sample and the detector to reduce the air scattering and absorption. The different tomographic projections were acquired using a binary acquisition strategy with 8 interlaced nests of projections as described by Kaestner *et al.* (2011). We acquired 800 projections within the angular range of 0° and 180° for the PC paste and 1300 projections for the PC-FA and PC-CC pastes. The dose imparted on the specimens during acquisition was estimated to range between 2.1×10^{7} Gy for the AFt phase in the neat PC sample to 4.5×10^{7} Gy for the C_3S phase in the PC-CC sample. For this estimation the surface dose was calculated as described in (Howells *et al.*, 2009) using the values for the attenuation coefficient and density resulting from the Avizo image analysis and detailed in Tables 3, 4 and 5- in the main article.

Ptychographic reconstructions were performed with a few hundred iterations of a difference map algorithm (Thibault, 2009) followed by a few hundred iterations of a maximum likelihood optimization used as a refinement (Thibault & Guizar-Sicairos, 2012). Before tomographic reconstruction, the phase projections were processed to remove zero and 1st linear terms, followed by phase unwrapping and vertical alignment (Guizar-Sicairos *et al.*, 2011). In addition, the horizontal alignment of the projections was performed using a tomographic consistency approach (Guizar-Sicairos *et al.*, 2015). The alignment corrections determined in this way were later also applied to the

amplitude images. Tomographic reconstructions were performed separately for the phase and amplitude projections to yield values of the refractive index decrement, δ(r), and the absorption index, i.e., the imaginary part, β(r), of the complex refractive index of the sample n(r) = 1- δ(r) + i β(r). For the amplitude tomogram we used filtered backprojection (FBP) with a Hanning filter with a 0.35 normalized cut-off frequency while for the phase tomogram we applied a modified FBP suitable for wrapped phase (Guizar-Sicairos *et al.*, 2011) using a Hanning filter with 1.0 normalized cut-off frequency, taking into account its higher signal-to-noise ratio.

Determination of densities and attenuation coefficients for the material phases.

The employed electron densities are those directly derived from the PXCT study, which were given in Tables 3,4 and 5 of the main text. In these tables, the δ values were converted to electron densities by using Eq. 1 (main text) and to mass densities by using Eq. 2 (main text). Ten different particles/volumes were independently analyzed for each phase and an average value was obtained. The errors given for electron densities in Tables 3, 4 and 5 are the standard deviations from the ten measurements. The δ values were also obtained from the segmentation volumes for each component by using Avizo® Fire edition v.8.0 software. The β values were converted to μ coefficients using Eq. 3 (main text) and they were reported in Tables 3, 4 and 5.

Determination of the average water content from PXCT data

Because the stoichiometry of $(CaO)_x(SiO_2)(H_2O)_y$ in the cement samples is not fully known, its density, ρ, cannot be directly determined through Equation [2] from the main text. We have followed the set of equations reported previously for determining the water content of C-S-H gel (da Silva *et al.*, 2015) in a tricalcium silicate paste:

$$\omega_{CaO} + \omega_{SiO_2} + \omega_{H_2O} = 1 \qquad \text{(S1)}$$

$$\frac{1}{x}\frac{\omega_{CaO}}{A_{CaO}} = \frac{\omega_{SiO_2}}{A_{SiO_2}} = \frac{1}{y}\frac{\omega_{H_2O}}{A_{H_2O}} \qquad \text{(S2)}$$

$$\omega_{CaO}\left(\frac{\mu}{\rho}\right)_{CaO} + \omega_{Al(OH)_3}\left(\frac{\mu}{\rho}\right)_{SiO_2} + \omega_{H_2O}\left(\frac{\mu}{\rho}\right)_{H_2O} = \frac{2\beta\ \lambda N_A r_o}{\delta}\left(\frac{xZ_{CaO}+Z_{SiO_2}\ yZ_{H_2O}}{xA_{CaO}+A_{SiO_2}+yA_{H_2O}}\right) \qquad \text{(S3)}$$

where λ is the X-ray wavelength, r_0 is the electron radius, NA is Avogrado's number, Z_i is the atomic number, A_i is the molar mass, β and δ are the measured imaginary part of the refractive index and real part of the refractive index decrement of the full material phase, respectively, $(\mu\cdot\rho^{-1})_i$ is the mass attenuation coefficient, and ω_i is the relative molar mass of the i-th material component, given by Cullity (1956) and Liu & Daum (2008)

$$\omega_i = \frac{a_i A_i}{\sum_j a_j A_j} \quad , \qquad \text{(S4)}$$

where i denotes each component, namely CaO, SiO_2 and H_2O, and j each atom in the component. The quantity to be determined is then aH_2O, which is denoted as y in Eqs. S2 and S3. For aCaO, the 1.8 value obtained from bibliography was fixed, denoted as x in Eqs. S2 and S3 and $aSiO_2$ is 1.

Finally, the same $\mu \cdot \rho^{-1}$values reported by da Silva et al. were used for this study, see S.I. in da Silva *et al.* (2015).

References

Cullity, B. D. (1956). *J. Chem. Educ.* **34**, A178.

Dinapoli, R., Bergamaschi, A., Henrich, B., Horisberger, R., Johnson, I., Mozzanica, A., Schmid, E., Schmitt, B., Schreiber, A., Shi, X. & Theidel, G. (2011). *Nucl. Instruments Methods Phys. Res. Sect. A Accel. Spectrometers, Detect. Assoc. Equip.* **650**, 79–83.

Edo, T. B., Batey, D. J., Maiden, A. M., Rau, C., Wagner, U., Pešić, Z. D., Waigh, T. A. & Rodenburg, J. M. (2013). *Phys. Rev. A*. **87**, 053850.

Gorelick, S., Vila-Comamala, J., Guzenko, V. A., Barrett, R., Salomé, M. & David, C. (2011). *J. Synchrotron Radiat.* **18**, 442–446.

Guizar-Sicairos, M., Boon, J. J., Mader, K., Diaz, A., Menzel, A. & Bunk, O. (2015). *Optica*. **2**, 259.

Guizar-Sicairos, M., Diaz, A., Holler, M., Lucas, M. S., Menzel, A., Wepf, R. A. & Bunk, O. (2011). *Opt. Express*. **19**, 21345.

Holler, M., Diaz, A., Guizar-Sicairos, M., Karvinen, P., Färm, E., Härkönen, E., Ritala, M., Menzel, A., Raabe, J. & Bunk, O. (2014). *Sci. Rep.* **4**, 3857.

Holler, M. & Raabe, J. (2015). **54**, 054101.

Holler, M., Raabe, J., Diaz, A., Quitmann, C., Menzel, A. & Bunk, O. (2012). **83**, 073703.

Howells, M. R., Beetz, T., Chapman, H. N., Cui, C., Holton, J. M., Kirz, J., Lima, E., Marchesini, S., Miao, H., Sayre, D., Shapiro, D. A., Spence, J. C. H. & Starodub, D. (2009). *J. Electron Spectros. Relat. Phenomena*. **170**, 4–12.

Huang, X., Yan, H., Harder, R., Hwu, Y., Robinson, I. K. & Chu, Y. S. (2014). *Opt. Express*. **22**, 12634.

Kaestner, A., Munch, B., Trtik, P. & Butler, L. (2011). *Opt. Eng.* **50**, 123201.

Liu, Y. & Daum, P. H. (2008). *J. Aerosol Sci.* **39**, 974–986.

Sánchez-Herrero, M. J., Fernández-Jiménez, A. & Palomo, Á. (2016). *J. Am. Ceram. Soc.* **99**, 604–611.

da Silva, J. C. & Menzel, A. (2015). *Opt. Express*. **23**, 33812.

da Silva, J. C., Trtik, P., Diaz, A., Holler, M., Guizar-Sicairos, M., Raabe, J., Bunk, O. & Menzel, A. (2015). *Langmuir*. **31**, 3779–3783.
